# An effective plasma membrane proteomics approach for small tissue samples

**DOI:** 10.1038/srep10917

**Published:** 2015-06-05

**Authors:** Katrien Smolders, Nathalie Lombaert, Dirk Valkenborg, Geert Baggerman, Lutgarde Arckens

**Affiliations:** 1 KU Leuven, Department of Biology, Laboratory of Neuroplasticity and Neuroproteomics, Leuven, Belgium; 2 Unit Environmental Risk & Health, VITO, Mol, Belgium; 3 Center for Proteomics, UAntwerp, Antwerp, Belgium; 4 Interuniversity Institute for Biostatistics and statistical Bioinformatics, Hasselt University, Belgium

## Abstract

Advancing the quest for new drug targets demands the development of innovative plasma membrane proteome research strategies applicable to small, functionally defined tissue samples. Biotinylation of acute tissue slices and streptavidin pull-down followed by shotgun proteomics allowed the selective extraction and identification of >1,600 proteins of which >60% are associated with the plasma membrane, including (G-protein coupled) receptors, ion channels and transporters, and this from mm^3^-scale tissue.

The plasma membrane (PM) physically separates a cell from its external environment and is composed out of a lipid bilayer and associated proteins^1^,^2^,^3^. The PM proteome is very dynamic because of extensive trafficking between the PM and the endomembrane compartment of eukaryotic cells via exocytosis, endocytosis and recycling processes^4^,^5^. PM proteins (PMPs) like (G-protein coupled) receptors, ion channels and transporters are crucial for a wide variety of fundamental physiological processes^6^. Targeted profiling of this PM proteome, and specifically the proteome exposed at the cell surface, is key to e.g. the identification of cell surface biomarkers or the isolation of tissue-specific cell types^2^,^7^,^8^,^9^. Their role in cell-cell interactions, molecular transport and signalling explains their potential as important therapeutic targets^1^,^10^,^11^.

PMPs exist in two main forms, the integral cell surface proteins spanning the lipid bilayer and the peripheral proteins, anchored to the PM^1^. This heterogeneity, the low overall abundance and hydrophobic nature, which results in poor solubility, few trypsin cleavage sites and difficult accessibility for proteases, make proteomic analysis of PMPs challenging^1^,^12^. Traditional isolation of PMPs from biological tissue samples by subcellular fractionation based on ultracentrifugation suffers from weak enrichment and contamination from other cellular compartments^1^,^7^. It also requires high sample loads, being a major disadvantage particularly in e.g. the field of neuroscience research where, usually, sample quantities are limited^6^,^13^.

It has been demonstrated that biotinylation of cell surface-exposed proteins followed by affinity purification from cell lines or cell cultures offers a usable alternative to the classical ultracentrifugation for the specific extraction and enrichment of PMPs^3^,^7^,^14^,^15^. In 2003, Thomas-Crusells and colleagues developed and optimized a comparable method for the biotinylation of such cell surface proteins in acute brain slices^16^. This, in combination with standard immunoblotting for predefined PMPs^17^,^18^,^19^, created the opportunity to study PMP trafficking in a more natural and physiologically relevant experimental setting^16^,^20^. Simultaneous *ex vivo* slice experiments such as electrophysiological recordings can be performed^16^. To our knowledge, biotinylation of acute tissue slices in conjunction with the proteomic profiling of the PM proteome has not yet been reported. Nevertheless, it holds the potential to solve both the problem of poor extraction efficiency and of high sample consumption characteristic to the more common tissue extraction protocols based on ultracentrifugation used in plasma membrane proteomics today.

## Results and Discussion

**Figure 1:**
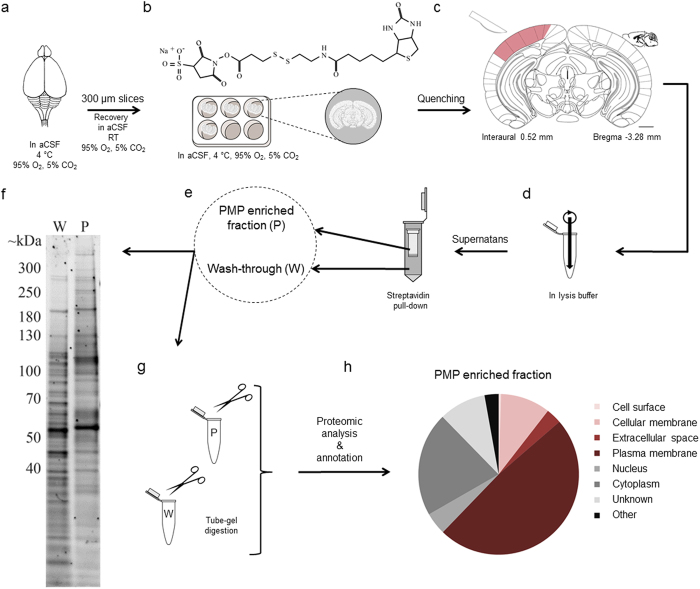
Workflow for plasma membrane proteomic analysis of small tissue samples. (**a**) Dissect organ of interest, like mouse brain, in artificial cerebrospinal fluid (aCSF). (**b**) Make slices, allow recovery, label with EZ-Link Sulfo-NHS-SS-Biotin. After quenching, dissect region of interest like the visual cortex (**c**, red) and mechanically homogenize (**d**). (**e**) Separate the plasma membrane protein (PMP) enriched fraction (P) from the rest of the proteome (wash-through, W) by streptavidin pull-down. Panel (**f**) illustrates SDS-PAGE for P and W. After digestion (**g**) analyse the protein samples and annotate (**h**). c adapted from^30^. Scale bar: 1 mm.

In this study, we performed an ‘acute slice biotinylation assay’ (ASBA) on mouse coronal brain slices (Fig. 1a–d) followed by streptavidin pull-down to separate cell surface-associated proteins in a subfraction termed the ‘PMP enriched fraction’, from the rest of the proteome termed the ‘wash-through fraction’ (Fig. 1e). Traditionally, biotinylation of acute slices and affinity purification is used in combination with immunoblotting to investigate trafficking of receptors and transporters in and out the PM in anatomically or functionally delineated regions of interest in a tissue^16^ such as mouse visual cortex in the forebrain^17^. With the intention to verify the applicability of ASBA in combination with proteomic analysis independent of a priori assumptions about the identity of PMPs of potential biological interest, and to a lot smaller tissue samples, we also isolated mouse visual cortex tissue (Fig. 1c; red), but on mm^3^-scale as study sample.

To judge the reproducibility of ASBA and streptavidin pull-down, a total protein stain was performed on 1 μg of proteins separated on SDS-PAGE belonging to the PMP enriched fractions and the wash-through fractions (Fig. 1f) derived from 5 different brain samples. The resulting pattern of protein bands, with a predominant location in the higher Mw regions, appeared identical for each of the 5 PMP enriched fractions and differed markedly from the pattern of protein bands, identical between all 5 wash-through fractions. For each of these protein samples we calculated the relative proportion of protein quantity in its PMP enriched fraction to the initial total protein content, that is the sum of the wash-through and PMP enriched fraction. The percentage of proteins in the PMP enriched fraction from each of the extracts ranged between 6.0 and 7.2%.

The clear dissimilarity in band pattern between a PMP enriched fraction and wash-through of one and the same ASBA extract (Fig. 1f), is indicative of a clear difference between the proteins retained on the Streptavidin agarose resin versus those in the eluent. This prompted us to identify the proteins present in the two fractions of each of the 5 brain samples using shotgun proteomics (Table 1 and Supplementary Data 1 and 2). An intermediate step of tube-gel digestion on 25 μg proteins per fraction improved solubilisation and digestion efficiency of membrane proteins^21^, and facilitated the removal of detergents prior to mass spectrometric analysis of 1 μg samples (Fig. 1g,h). Next, we used IPA to categorize each identified protein present in the 5 PMP enriched fractions into their subcellular compartment, as an extra validation of the capability of the ASBA method to truly enrich PMPs from a proteomic sample. The percentage of proteins categorised as PMPs by IPA in the 5 separate PMP enriched fractions ranged between 26.8 and 28.8%, illustrating enrichment in and reproducibility of our workflow (Table 1).

For a more detailed analysis of the plasma membrane proteome, we then merged all identifications of the 5 PMP enriched fractions together into 1 protein list. We also merged the identification lists of the 5 wash-through fractions. This resulted in respectively 1,698 and 2,872 discrete proteins that were identified in that PMP enriched fraction and wash-through fraction (Table 1). Of these 1,698 proteins identified in this PMP enriched fraction, IPA successfully annotated 1,625 proteins. Out of these 1,625, 417 proteins or 25.7% were classified as PMP (Table 1 and 2). Because IPA only provides one subcellular localization per protein and does not consider the additional cellular compartments in which a protein can occur^15^, secondary annotations were also checked in IPA and DAVID, in combination with an intensive literature search^6^. As such, a large number of proteins (372) could be additionally assigned to potentially reside in association with (peripheral proteins) or even be fully embedded within the plasma membrane (integral proteins), leading to a total of 789 or 48.6% of PMPs in the PMP enriched fraction (Table 2, Supplementary Table 1, Fig. 1h). Of note, this additional analysis classified an even larger subset of PMPs as ion channel, transmembrane receptor, transporter or G-protein coupled receptor (Table 2 and Supplementary Table 1). Together with these 789 annotated PMPs, another 8 proteins located at the cell surface (0.5%), 163 at the cellular membrane (10.0%) and 51 proteins in the extracellular space (3.1%) (Supplementary Table 2), this accounts for 1,011 or 62.2% proteins in the PMP enriched fraction that have been reported to reside in or near the cell surface (Fig. 1h). The remaining 37.8% proteins in the PMP enriched fraction might be the result of co-purification of large intracellular complexes, with the biotinylated proteins still associated to the plasma membrane or with the readily releasable vesicle pools. These proteins deserve attention in future research to either confirm or exclude their capacity to potentially reside at the PM. In sum, our yields are in agreement with or even higher than recent PMP enrichment studies based on aqueous two-phase affinity partitioning of a much larger tissue sample, a complete rat or mouse cerebellum^13^,^22^, or on biotinylation and affinity purification of cell surface proteins of cultured mouse cortical neurons^14^.

The efficiency of the presented workflow for the enrichment of PMPs was further validated using the DAVID web tool. This tool allows visualization of the specific protein enrichment in the PMP enriched and wash-through fractions by a gene ontology enrichment analysis on all protein identifications of each fraction relative to a background. The background was built by merging all proteins identified in the PMP enriched with those from the wash-through fraction into one background data set. We used the functional annotation charts of the DAVID web tool based on cellular component ontology and visualized the results in ReViGO treemaps (Supplementary Fig. 1 and 2). The treemap adapted from ReViGO for the PMP enriched fraction (Supplementary Fig. 1) is summarized in Table 3, illustrating an enrichment of proteins associated to the cell surface specific for neuronal cells in the clusters ‘plasma membrane’, ‘plasma membrane part’, ‘ion channel complex’, ‘intrinsic’ and ‘integral component of PM’, and the clusters ‘synapse’, ‘synapse part’, ‘neuron projection’, ‘dendritic spine’, and ‘cell junction’. The treemap adapted from ReViGO resulting from our wash-through data set (Supplementary Fig. 2) summarized in Table 4 shows clusters of proteins associated with different intracellular organelles, especially with mitochondrial function and the ‘respiratory chain’. This reflects the high energy demand and oxygen consumption of neurons, and thus the high metabolic rate of the tissue under study^23^,^24^,^25^,^26^,^27^. Other clusters contain proteins with a role at the envelope, within the endomembrane system, and within the membrane-enclosed lumen. Importantly, no clear cluster was suggested for cell surface-associated proteins for the wash-through fraction.

In conclusion, in this report we present the new combination of a procedure for the specific extraction of cell surface-associated proteins including PMPs originating from mm^3^-scale tissue derived from acute tissue slice preparations, with proteomic analysis. This reproducible and efficient enrichment methodology is undoubtedly applicable to many different tissue types and can significantly contribute to future differential plasma membrane proteomics research in many fields of application ranging from neuroscience, cancer, and immunology, to stem cell research.

## Methods

### Animals

Adult (n = 5) C57Bl/6J mice of either sex were housed under standard laboratory conditions with a daily photoperiod of 13 hours light and 11 hours darkness with water and food available *ad libitum*.

All experiments were approved by the ethical research committee of KU Leuven and were in strict accordance with the European Communities Council Directive of 22 September 2010 (2010/63/EU) and with the Belgian legislation (KB of 29 May 2013). Every possible effort was made to minimize animal suffering and to reduce the numbers of animals.

### Acute slice biotinylation assay (ASBA)

Mice were killed by cervical dislocation. Brains were rapidly dissected in ice-cold (4 °C) artificial cerebrospinal fluid (aCSF; 124 mM NaCl, 4.9 mM KCl, 2 mM MgSO_4_, 2 mM CaCl_2_, 1.2 mM KH_2_PO_4_, 25.6 mM NaHCO_3_, and 10 mM D-glucose; pH 7.4) saturated with 95% O_2_ and 5% CO_2_. Subsequently, each brain was separated in half along the longitudinal fissure, and the left hemisphere was cut into 300 μm-thick coronal slices using a Vibratome (HM 650 V, Prosan). A thickness of tissue slices between 300 and 400 μm is essential to biotinylate an amount of functionally healthy and intact cells that will outnumber the sliced ones^28^,^29^. Four slices between Bregma level –2.70 and –4.16 of each hemisphere were placed in an incubation chamber (65-0076/BSC-PC, Harvard Apparatus by Warner Instruments) filled with aCSF and provided with a continuous flow of 95% O_2_ and 5% CO_2_ for 90 min in order to recover.

Next, the sections were put on ice and supplemented with CO_2_ and O_2_ throughout the whole biotinylation procedure. They were washed twice in ice-cold aCSF, incubated for 45 min with EZ-Link Sulfo-NHS-SS-Biotin (0.5 mg ml^−1^ in aCSF; Thermo Scientific) and washed twice with ice-cold aCSF complemented by 100 μM lysine (Sigma) to block the remaining reactive Sulfo-NHS-SS-biotin. It has already been demonstrated that these tissue slices stay viable with the cells intact during the complete process. Incubation on ice during biotin labelling will limit protein internalization to a minimum in order to create a snapshot of the cell surface proteome. The Sulfo-NHS-SS-biotin can reach all cell layers of a slice up to 350 μm-thick after incubation with minimum 100 μM for at least 45 min. Because of the intact cell membranes and the membrane impermeability of the biotin label, cell surface-exposed proteins will be biotinylated and labelling of intracellular proteins will be minimal^16^, as substantiated with our approach and data set.

The sections were washed and kept in ice-cold, saturated aCSF until dissection of the region of interest under a binocular microscope (ASZ30E; Bausch & Lomb). The borders of the visual cortex were determined based on the stereotaxic mouse brain atlas^30^. For each brain, the dissected visual cortex tissue from the four slices was collected in 100 μL lysis buffer (1% Triton X-100, 0.1% sodium dodecyl sulphate (SDS), 1 mM ethylenediaminetetraacetic acid (EDTA), 50 mM NaCl, 20 mM Tris; pH 7.5) and 4 μl of a mix of protease inhibitors (Roche). After mechanical homogenization and centrifugation (10,000g, 5 min, 4 °C) the supernatant was collected and stored at –80 °C. The total protein concentration of the 5 biotin-labelled samples was determined according to the Qubit^TM^ Quantification Platform (Invitrogen) using a Qubit^TM^ fluorometer (Invitrogen, Merelbeke, Belgium).

### Isolation of plasma membrane proteins

Biotin-labelled proteins were separated from the rest of the proteome by a protocol based on biotin’s affinity for streptavidin. For this purpose, 150 μl of at room temperature (RT) calibrated Streptavidin agarose resin (Thermo Scientific) was loaded onto a Pierce^®^ Spin Cups - Cellulose Acetate Filter (Thermo Scientific). After centrifugation, the column was centrifuged (500 g, 1 min) and washed four times by adding 700 μl of phosphate-buffered saline (PBS; 0.1 M H_3_PO_4_, 0.15 M NaCl; pH 7.2) and centrifugation (500 g, 1 min). The 5 biotinylated samples, each containing ±500 μg of proteins extracted from ±1 mm^3^ tissue, were loaded onto 5 prepared columns and all were shaken for 15 min at RT. After centrifugation (500 g, 1 min), the columns were washed three times with 700 μl PBS. All of the non-biotinylated proteins were eluted during the wash steps with the vast majority of proteins eluted in the first wash-through. Next, 5 μl 2% SDS, 45 μl 200 mM Triethylammonium bicarbonate (TEAB; Fluka Analytical), 45 μl MilliQ (Merck Millipore) and 5 μl 200 mM Tris (2-carboxyethyl) phosphine (TCEP; Thermo Scientific) were added to each column and they were incubated for 1 h at 55 °C for denaturation and reduction. Samples were alkylated for 30 min in the dark in 5 μl 375 mM iodoacetamide (IAA; Thermo Scientific), centrifuged (500 g, 1 min) and the 5 obtained eluents were collected. Subsequently, 25 μl 200 mM TEAB and 25 μl MilliQ were added to each column, they were centrifuged (500 g, 1 min), each eluent added to the corresponding previous one and these 5 combined samples, being the plasma membrane protein (PMP) enriched fractions, were stored at –20 °C, together with the 5 first wash-through fractions.

### Protein quantity determination

The total protein concentration of the first wash-throughs and the PMP enriched fractions, were determined according to the Qubit^TM^ Quantification Platform (Invitrogen) as described above. For each sample, the relative proportion of protein quantity in the PMP enriched fraction was calculated to the total protein content of the initial sample, the wash-through and PMP enriched fraction combined. For the calculation of these percentages, original Qubit protein quantitation measurements were used and renumbered according to the total volume of each fraction.

### Gel-electrophoresis and total protein stain

To 1 μg protein of each of the wash-throughs and 1 μg protein of each of the PMP enriched fractions, 5 μl of XT Sample Buffer solution (4x; Bio-Rad) and 1 μl of XT Reducing Agent (20x; Bio-Rad) was added. The protein samples were denatured and reduced for 10 min on 70 °C. Proteins were separated on a Criterion^TM^ Precast Gel^XT^ 4–12% Bis-Tris (Bio-Rad) in the Criterion^TM^ Cell (Bio-Rad) system and 4 μL of the Spectra^TM^ Multicolor High range protein ladder (Thermo Scientific) was used as molecular weight standard. Next, we performed a total protein stain with Serva Purple (SERVA) according to manufacturer’s instructions. After the staining, the gel was scanned with the Bio-Rad ChemiDoc^TM^ MP Imaging System.

### Proteomic analysis

For the 5 wash-throughs and the 5 PMP enriched fractions, 25 μg was diluted in MilliQ to a total volume of 100 μL. The samples were subsequently transformed into tube-gels by adding 25 μL of Acrylamide/Bis solution (40% 29:1; Bio-Rad), 1 μl SDS (10%), 0.5 μl APS (ammonium persulfate, 10%; Sigma) and 0.1 μl Temed (N, N, N’, N’ - Tetramethylethylenediamine; Fluka BioChemika) and incubation of 30 min at RT. Peptides were extracted from these tube-gels by in-gel trypsin digestion. The tube gels were cut in pieces of approximately 1 mm^3^. The pieces were washed twice with 50 μl MilliQ, followed by 3 × 50 μl acetonitrile. After three cycles of hydration with acetonitrile and rehydration with 100 mM ammonium bicarbonate, the gel pieces were vacuum dried in a vacuum concentrator. To start the enzymatic digestion, 25 μl of a solution containing 5 ng μl^−1^ trypsin (Promega), 50 mM ammonium bicarbonate and 5 mM calciumchloride was added to each gel piece and placed on 37 °C overnight. The next day, the tryptic peptides were extracted using 50 mM ammonium bicarbonate followed by an extraction with 50% acetonitrile and 5% formic acid. This step was repeated twice. Afterwards, the pooled extracts were vacuum dried and the peptides were stored at –20 °C. The equivalent of 1 μg of total protein was loaded and analysed by nanoLC-mass spectrometry. Liquid chromatography mass spectrometric analysis was performed on a Waters nanoAquity LC system connected to a Thermo Scientific LTQ Velos Orbitrap mass spectrometer. The equivalent of 2 μg of total protein of the digested sample was dissolved in 20 μl of 2% acetonitrile in HPLC-grade water. 10 μl of the sample was loaded onto the trapping column (Pepmap C18 300 μm x 20 mm, Dionex) with an isocratic flow of 2% acetonitrile in water with 0.1% formic acid at a flow rate of 5 μl min^−1^. After 2 min, the column-switching valve was switched, placing the pre-column online with the analytical capillary column, a Pepmap C18, 3 μm 75 μm x 150 mm nano column (Dionex). Separation was conducted using a linear gradient from 2% acetonitril in water, 0.1% formic acid to 40% acetonitril in water, 0.1% formic acid in 160 min. The flow rate was set at 400 nl min^−1^. The LTQ Orbitrap Velos (Thermo Scientific) was set up in a data dependent MS/MS mode where a full scan spectrum (350–5,000 m/z, resolution 60,000) was followed by a maximum of ten CID tandem mass spectrum (100 to 2,000 m/z). Peptide ions were selected as the twenty most intense peaks of the MS1 scan. Collision induced dissociation (CID) scans were acquired in the LTQ iontrap part of the mass spectrometer. The normalized collision energy used was 35% in CID. We applied a dynamic exclusion list of 45 s.

### Data analysis

Proteome Discoverer 1.3.0.339 (Thermo Scientific) was used as a workflow manager to handle the data. The tandem MS data were searched using both SEQUEST and Mascot (Matrix science) against the Swissprot database (v 09/2012, 538010 sequences) for *Mus musculus* taxonomy. All tandem mass spectra in the range of 300 Da to 8,000 Da were interpreted.

Monoisotopic peak assignment, charge state determination, co-isolation interference, and delta mass calculation between the measured and theoretical monoisotopic masses were determined by Proteome Discoverer. The precursor mass tolerance was set at 5 ppm, while fragment mass tolerance was set to 0.5 Da. A maximum of two missed cleavages by trypsine was allowed for. A static modification of 57.021 Da on cysteine was defined to allow for carbamidomethylation. Further, a dynamic modification of 15.9955 Da was introduced to account for possible oxidation of methionine. The use of average precursor masses and average fragment masses was prohibited. Only first ranked PSMs were considered for further analysis. The false discovery rate is controlled by a target-decoy approach on a reversed database. Peptide spectrum matches were found significant at an FDR of 5%.

Protein discoverer 1.3.0.339 was used to combine the significant peptide annotation from Mascot and SEQUEST in a parsimonious protein list.

Protein grouping follows the rule of parsimony. Essentially only the minimal list of proteins that can explain all the peptides in the data set is reported.

Ingenuity Pathway Analyis (IPA^®^ , QIAGEN Redwood City, www.qiagen.com/ingenuity) and Database of Annotation, Visualization and Integrated Discovery (DAVID, version 6.7) were used for cellular component assignment.

The enrichment of proteins in the PMP enriched fraction was investigated by analysing all identifications within this fraction relative to a background composed of all proteins identified in the wash-through and PMP enriched fraction together with DAVID^31^,^32^. DAVID summarized the cellular component ontology identified via a functional annotation chart and calculated a Fisher exact test for each ontology as a measure for enrichment within the fraction. Similarly, information about the enrichment of proteins in the wash-through fraction was retrieved. The GO-terms and corresponding p-values with Benjamini correction were subsequently submitted to ReViGO, a web server that Reduces and Visualizes long lists of Gene Ontology terms^33^, and visualized in treemaps that cluster ontologies with high semantic similarity. The size of these cluster representatives, which are joined in different superclusters each indicated by 1 colour, is proportional to the p-values derived by ReViGO. The treemap figures were then adapted by changing the colours of the superclusters.

## Additional Information

**How to cite this article**: Smolders, K. *et al.* An effective plasma membrane proteomics approach for small tissue samples. *Sci. Rep.* **5**, 10917; doi: 10.1038/srep10917 (2015).

## Supplementary Material

Supplementary Information

Supplementary Information

Supplementary Information

## Figures and Tables

**Table 1:** Percentage of PMPs in the 5 PMP enriched fractions.

**sample**	**# of ≠ IDs**	**# of ≠ IDs annotated by IPA**	**# of PMPs (1° IPA)**	**% of PMPs**	
**PMP ENRICHED FRACTION**					
1	968	934	250	26.8	
2	927	896	242	27.0	
3	872	846	244	28.8	
4	922	890	254	28.5	
5	993	968	270	27.9	
merge 1,2,3,4,5	1,698	1,625	417	25.7	
					
**WASH-THROUGH FRACTION**					
1	1,594				
2	1,587				
3	1,640				
4	1,494				
5	1,584				
merge 1,2,3,4,5	2,872				

**Table 2:** 417 PMPs in the PMP enriched fraction (1° IPA annotation).

**Accession no.**	**Protein name**	**identified in # of samples**
**ION CHANNEL**		
IPI00113149	syntaxin 1B	5
IPI00113244	tweety family member 1	4
IPI00113772	gamma-aminobutyric acid (GABA) A receptor, alpha 1	5
IPI00122300	calcium channel, voltage-dependent, gamma subunit 3	5
IPI00122974	glycoprotein M6A	5
IPI00129491	potassium voltage-gated channel, Shal-related subfamily, member 2	4
IPI00129774	potassium voltage-gated channel, shaker-related subfamily, member 2	5
IPI00130253	calcium channel, voltage-dependent, alpha 2/delta subunit 3	5
IPI00130546	gamma-aminobutyric acid (GABA) A receptor, beta 3	5
IPI00136965	glutamate receptor, ionotropic, AMPA 1	5
IPI00315359	potassium voltage-gated channel, shaker-related subfamily, beta member 2	4
IPI00322698	transient receptor potential cation channel, subfamily V, member 2	5
IPI00323554	gamma-aminobutyric acid (GABA) A receptor, beta 2	5
IPI00338309	ryanodine receptor 2 (cardiac)	5
IPI00410982	calcium channel, voltage-dependent, alpha 2/delta subunit 1	5
IPI00461322	annexin A7	5
IPI00608056	glutamate receptor, ionotropic, N-methyl D-aspartate 1	5
IPI00625961	calcium channel, voltage-dependent, P/Q type, alpha 1A subunit	1
IPI00652101	potassium large conductance calcium-activated channel, subfamily M, alpha member 1	5
IPI00673613	sodium channel, voltage-gated, type I, alpha subunit	1
IPI00751228	glutamate receptor, ionotropic, AMPA 2	5
IPI00761641	sodium channel, voltage-gated, type II, alpha subunit	5
IPI00877256	tweety family member 3	5
IPI00895035	glutamate receptor, ionotropic, AMPA 3	5
IPI00930821	potassium channel tetramerization domain containing 12	2
IPI01019157	potassium channel tetramerization domain containing 12	2
IPI00110601	gamma-aminobutyric acid (GABA) A receptor, alpha 3	3
IPI00119283	gamma-aminobutyric acid (GABA) A receptor, beta 1	1
IPI00119615	potassium inwardly-rectifying channel, subfamily J, member 3	1
IPI00120318	glutamate receptor, ionotropic, kainate 3	1
IPI00128826	calcium channel, voltage-dependent, gamma subunit 8	4
IPI00130455	FXYD domain containing ion transport regulator 1	4
IPI00131471	glutamate receptor, ionotropic, AMPA 4	1
IPI00132786	calcium channel, voltage-dependent, gamma subunit 2	1
IPI00133980	hyperpolarization activated cyclic nucleotide-gated potassium channel 2	1
IPI00228358	gamma-aminobutyric acid (GABA) A receptor, gamma 2	3
IPI00331064	calcium channel, voltage-dependent, R type, alpha 1E subunit	1
IPI00421206	potassium channel tetramerization domain containing 12	3
IPI00473235	calcium channel, voltage-dependent, beta 4 subunit	3
IPI00554917	chloride channel, voltage-sensitive 6	1
IPI00625414	calcium channel, voltage-dependent, N type, alpha 1B subunit	1
IPI00751689	calcium channel, voltage-dependent, alpha 2/delta subunit 2	1
IPI00752080	integrin, alpha V	4
IPI00775995	calcium channel, voltage-dependent, L type, alpha 1F subunit	1
IPI00844657	potassium voltage-gated channel, Shaw-related subfamily, member 3	1
IPI00874658	gamma-aminobutyric acid (GABA) A receptor, gamma 2	1
IPI00875552	tweety family member 1	1
IPI00928524	potassium voltage-gated channel, shaker-related subfamily, beta member 2	1
IPI00930809	calcium channel, voltage-dependent, gamma subunit 3	1
		
**TRANSMEMBRANE RECEPTOR**		
IPI00114939	neuronal pentraxin receptor	5
IPI00119063	low density lipoprotein receptor-related protein 1	5
IPI00137311	plexin A1	5
IPI00229992	plexin B1	5
IPI00403079	CD47 molecule	5
IPI00462790	coagulation factor III (thromboplastin, tissue factor)	4
IPI00463489	opioid binding protein/cell adhesion molecule-like	5
IPI00473582	ciliary neurotrophic factor receptor	5
IPI00756275	plexin B2	2
IPI00876097	plexin A4	5
IPI00130995	interleukin 18 receptor accessory protein	1
IPI00313025	scavenger receptor class A, member 3	1
IPI00315280	semaphorin 7A, GPI membrane anchor (John Milton Hagen blood group)	1
IPI00351062	cholinergic receptor, nicotinic, alpha 9 (neuronal)	1
IPI00463026	interleukin 1 receptor accessory protein-like 1	1
IPI00471022	plexin D1	4
IPI00480518	sema domain, transmembrane domain (TM), and cytoplasmic domain, (semaphorin) 6A	1
IPI00674255	plexin C1	2
IPI00754710	leukocyte immunoglobulin-like receptor, subfamily B (with TM and ITIM domains), member 3	1
IPI00874523	roundabout, axon guidance receptor, homolog 1 (Drosophila)	2
IPI00986716	plexin B2	2
IPI00989396	roundabout, axon guidance receptor, homolog 1 (Drosophila)	1
		
**TRANSPORTER**		
IPI00109153	solute carrier family 17 (vesicular glutamate transporter), member 7	5
IPI00111151	rabphilin 3A	5
IPI00113869	basigin (Ok blood group)	5
IPI00114279	solute carrier family 1 (glial high affinity glutamate transporter), member 3	5
IPI00114641	solute carrier family 3 (amino acid transporter heavy chain), member 2	5
IPI00121550	ATPase, Na+/K+transporting, beta 1 polypeptide	5
IPI00122048	ATPase, Na+/K+transporting, alpha 3 polypeptide	5
IPI00123704	ATPase, Na+/K+transporting, beta 2 polypeptide	5
IPI00124221	ATPase, Na+/K+transporting, beta 3 polypeptide	2
IPI00125397	solute carrier family 30 (zinc transporter), member 3	1
IPI00125635	synaptosomal-associated protein, 25 kDa	5
IPI00126796	solute carrier family 27 (fatty acid transporter), member 4	3
IPI00127713	ATPase, Ca++transporting, plasma membrane 2	5
IPI00134191	solute carrier family 2 (facilitated glucose transporter), member 3	5
IPI00135130	solute carrier family 1 (glutamate/neutral amino acid transporter), member 4	5
IPI00136372	synapsin I	5
IPI00137194	solute carrier family 16 (monocarboxylate transporter), member 1	1
IPI00221456	synaptic vesicle glycoprotein 2B	5
IPI00227928	solute carrier family 6 (neurotransmitter transporter), member 1	4
IPI00230289	solute carrier family 1 (glial high affinity glutamate transporter), member 2	1
IPI00268433	solute carrier family 8 (sodium/calcium exchanger), member 1	3
IPI00308691	solute carrier family 2 (facilitated glucose transporter), member 1	5
IPI00311682	ATPase, Na+/K+transporting, alpha 1 polypeptide	5
IPI00314289	solute carrier family 1 (neuronal/epithelial high affinity glutamate transporter, system Xag), member 1	4
IPI00322156	solute carrier family 38, member 3	5
IPI00331577	solute carrier family 7 (amino acid transporter light chain, L system), member 5	4
IPI00403860	neurexin 1	5
IPI00407692	ATPase, H+transporting, lysosomal 70 kDa, V1 subunit A	5
IPI00420244	solute carrier family 1 (neuronal/epithelial high affinity glutamate transporter, system Xag), member 1	4
IPI00420569	ATPase, Na+/K+transporting, alpha 2 polypeptide	5
IPI00465769	solute carrier family 12 (potassium/chloride transporter), member 5	5
IPI00555118	solute carrier family 4, sodium bicarbonate transporter, member 10	5
IPI00556827	ATPase, Ca++transporting, plasma membrane 1	5
IPI00621162	ATPase, Ca++transporting, plasma membrane 3	3
IPI00648537	solute carrier family 24 (sodium/potassium/calcium exchanger), member 2	3
IPI00648633	solute carrier family 44 (choline transporter), member 1	1
IPI00750917	ATPase, Ca++transporting, plasma membrane 4	2
IPI00754989	solute carrier family 39 (zinc transporter), member 12	5
IPI00857092	solute carrier family 4 (sodium bicarbonate cotransporter), member 4	5
IPI00884508	solute carrier family 2 (facilitated glucose transporter), member 1	5
IPI00890144	solute carrier family 4, sodium bicarbonate cotransporter, member 8	5
IPI00970455	neurexin 1	5
IPI00125830	Ly6/neurotoxin 1	2
IPI00128152	ATP-binding cassette, sub-family B (MDR/TAP), member 1B	3
IPI00128391	megalencephalic leukoencephalopathy with subcortical cysts 1	1
IPI00129395	solute carrier family 7 (amino acid transporter light chain, L system), member 5	1
IPI00135632	solute carrier family 7 (amino acid transporter light chain, L system), member 8	1
IPI00135678	X-linked Kx blood group	1
IPI00136867	solute carrier family 6 (neurotransmitter transporter), member 11	3
IPI00153278	solute carrier family 29 (equilibrative nucleoside transporter), member 4	1
IPI00165688	solute carrier family 23 (ascorbic acid transporter), member 2	2
IPI00170146	ATP-binding cassette, sub-family A (ABC1), member 6	1
IPI00172274	ATP-binding cassette, sub-family C (CFTR/MRP), member 10	1
IPI00221831	solute carrier family 32 (GABA vesicular transporter), member 1	4
IPI00221932	mal, T-cell differentiation protein 2 (gene/pseudogene)	3
IPI00230290	solute carrier family 1 (glial high affinity glutamate transporter), member 2	4
IPI00310247	ANKH inorganic pyrophosphate transport regulator	2
IPI00338618	ATPase, class V, type 10A	1
IPI00380273	gap junction protein, alpha 1, 43 kDa	3
IPI00463589	ATPase, Ca++transporting, plasma membrane 4	3
IPI00623542	solute carrier family 8 (sodium/calcium exchanger), member 1	2
IPI00648270	solute carrier family 44 (choline transporter), member 1	4
IPI00652257	solute carrier family 24 (sodium/potassium/calcium exchanger), member 2	1
IPI00776182	solute carrier family 9, subfamily A (NHE6, cation proton antiporter 6), member 6	1
IPI00785299	ATPase, Ca++transporting, plasma membrane 3	2
IPI00788403	ATP-binding cassette, sub-family A (ABC1), member 8	1
IPI00927968	copine VI (neuronal)	2
		
**G-PROTEIN COUPLED RECEPTOR**		
IPI00132061	purinergic receptor P2Y, G-protein coupled, 12	3
IPI00135659	oligodendrocyte myelin glycoprotein	5
IPI00136716	glutamate receptor, metabotropic 3	5
IPI00229528	brain-specific angiogenesis inhibitor 1	4
IPI00281619	glutamate receptor, metabotropic 1	4
IPI00407689	gamma-aminobutyric acid (GABA) B receptor, 1	3
IPI00465871	G protein-coupled receptor 158	5
IPI00762862	glutamate receptor, metabotropic 2	5
IPI00816879	latrophilin 1	5
IPI00881441	latrophilin 3	3
IPI01018412	gamma-aminobutyric acid (GABA) B receptor, 1	3
IPI00117887	neuromedin B receptor	1
IPI00120115	sphingosine-1-phosphate receptor 1	1
IPI00126064	olfactory receptor 1018	1
IPI00127181	olfactory receptor 1	1
IPI00136713	olfactory receptor 157	1
IPI00153507	vomeronasal 1 receptor 217	1
IPI00229361	glutamate receptor, metabotropic 6	1
IPI00269278	G protein-coupled receptor 119	1
IPI00402890	adenylate cyclase activating polypeptide 1 (pituitary) receptor type I	1
IPI00470960	glutamate receptor, metabotropic 4	2
IPI00474802	glutamate receptor, metabotropic 7	2
IPI00553387	glutamate receptor, metabotropic 5	2
IPI00605298	G protein-coupled receptor 123	1
IPI00675087	vomeronasal 2, receptor 32	1
IPI00755301	gamma-aminobutyric acid (GABA) B receptor, 1	2
IPI00867815	glutamate receptor, metabotropic 5	3
IPI00880691	latrophilin 3	2
IPI00944116	adenosine A3 receptor	1
		
**KINASE**		
IPI00125147	membrane protein, palmitoylated 2 (MAGUK p55 subfamily member 2)	5
IPI00129198	EPH receptor A4	5
IPI00314316	membrane protein, palmitoylated 6 (MAGUK p55 subfamily member 6)	4
IPI00337992	EPH receptor A4	5
IPI00351246	membrane protein, palmitoylated 3 (MAGUK p55 subfamily member 3)	5
IPI00626797	discs, large homolog 4 (Drosophila)	5
IPI00672505	discs, large homolog 1 (Drosophila)	5
IPI00762272	discs, large homolog 2 (Drosophila)	5
IPI00776413	calcium/calmodulin-dependent serine protein kinase (MAGUK family)	2
IPI00830221	EPH receptor B4	2
IPI00830635	EPH receptor A5	3
IPI00128360	neurotrophic tyrosine kinase, receptor, type 2	4
IPI00229334	neurotrophic tyrosine kinase, receptor, type 2	1
IPI00338094	bone morphogenetic protein receptor, type II (serine/threonine kinase)	1
IPI00474411	TYRO3 protein tyrosine kinase	5
IPI00474965	epidermal growth factor receptor	3
IPI00655218	phosphatidylinositol-4-phosphate 5-kinase, type I, gamma	1
IPI00808241	EPH receptor B1	2
IPI00875987	G protein-coupled receptor kinase 6	1
IPI00886325	membrane protein, palmitoylated 1, 55 kDa	1
IPI00918777	phosphatidylinositol-4-phosphate 5-kinase, type I, gamma	1
		
**PEPTIDASE**		
IPI00627016	ADAM metallopeptidase domain 22	5
IPI00650001	ADAM metallopeptidase domain 23	4
IPI00798468	ubiquitin specific peptidase 9, X-linked	4
IPI00881709	dipeptidyl-peptidase 6	5
IPI00118674	nicastrin	3
IPI00169524	thyrotropin-releasing hormone degrading enzyme	1
IPI00408232	ADAM metallopeptidase domain 11	2
IPI00621146	transmembrane protease, serine 11c	1
IPI00648033	ADAM metallopeptidase domain 23	1
IPI00752133	signal peptide peptidase like 2A	1
IPI00928374	nicastrin	2
IPI01027504	ubiquitin specific peptidase 9, X-linked	1
IPI01027684	ubiquitin specific peptidase 9, X-linked	1
		
**PHOSPHATASE**		
IPI00115626	phosphatidic acid phosphatase type 2B	2
IPI00405703	protein tyrosine phosphatase, receptor type, A	1
IPI00420590	lipid phosphate phosphatase-related protein type 4	5
IPI00465836	protein tyrosine phosphatase, receptor type, D	5
IPI00627008	protein tyrosine phosphatase, receptor-type, Z polypeptide 1	5
IPI00875821	SET binding factor 1	5
IPI00876489	signal-regulatory protein alpha	5
IPI00915502	protein tyrosine phosphatase, receptor type, S	5
IPI01027153	protein tyrosine phosphatase, receptor type, D	5
IPI00110264	protein tyrosine phosphatase, receptor type, F	1
IPI00336550	protein tyrosine phosphatase, receptor type, A	2
IPI00399905	protein tyrosine phosphatase, receptor type, f polypeptide (PTPRF), interacting protein (liprin), alpha 3	1
IPI00475109	protein phosphatase 3, catalytic subunit, beta isozyme	4
IPI00857748	protein tyrosine phosphatase, receptor type, f polypeptide (PTPRF), interacting protein (liprin), alpha 3	3
IPI00881167	protein tyrosine phosphatase, receptor type, G	1
		
**TRANSCRIPTION REGULATOR**		
IPI00222057	neogenin 1	3
		
**ENZYME**		
IPI00115429	gamma-glutamyltransferase 7	5
IPI00117176	fatty acid amide hydrolase	1
IPI00120716	guanine nucleotide binding protein (G protein), beta polypeptide 1	5
IPI00121387	guanine nucleotide binding protein (G protein), alpha 11 (Gq class)	4
IPI00123058	contactin 1	5
IPI00126551	DIRAS family, GTP-binding RAS-like 2	5
IPI00130949	adenylate cyclase 1 (brain)	5
IPI00133218	ADP-ribosylation factor-like 8B	3
IPI00138716	RAP2B, member of RAS oncogene family	5
IPI00162780	guanine nucleotide binding protein (G protein), beta polypeptide 2	1
IPI00222125	catechol-O-methyltransferase domain containing 1	2
IPI00228618	guanine nucleotide binding protein (G protein), q polypeptide	5
IPI00230192	guanine nucleotide binding protein (G protein), alpha activating activity polypeptide O	4
IPI00230193	guanine nucleotide binding protein (G protein), alpha z polypeptide	5
IPI00230194	guanine nucleotide binding protein (G protein), gamma 2	2
IPI00309113	neuroligin 1	4
IPI00378017	guanine nucleotide binding protein (G protein), beta 5	5
IPI00396701	RAP2A, member of RAS oncogene family	5
IPI00467152	guanine nucleotide binding protein (G protein), alpha inhibiting activity polypeptide 1	5
IPI00468605	neuroligin 2	5
IPI00649388	guanine nucleotide binding protein (G protein), alpha 13	2
IPI00816946	gephyrin	4
IPI00858047	monoglyceride lipase	5
IPI00881278	adenylate cyclase 9	5
IPI00928550	gamma-glutamyltransferase 7	5
IPI00929787	trans-2,3-enoyl-CoA reductase	5
IPI00115546	guanine nucleotide binding protein (G protein), alpha activating activity polypeptide O	1
IPI00123623	hyaluronan synthase 1	1
IPI00126501	carbonic anhydrase XIV	5
IPI00128097	adenylate cyclase 4	1
IPI00225670	gephyrin	1
IPI00228295	contactin 4	1
IPI00272230	RAB39B, member RAS oncogene family	1
IPI00315334	neuroblastoma RAS viral (v-ras) oncogene homolog	1
IPI00331267	ABO blood group (transferase A, alpha 1-3-N-acetylgalactosaminyltransferase; transferase B, alpha 1-3-galactosyltransferase)	1
IPI00377311	diacylglycerol lipase, alpha	1
IPI00403586	neutral cholesterol ester hydrolase 1	1
IPI00649078	SH3-domain GRB2-like 2	2
IPI00652606	RAB2B, member RAS oncogene family	2
IPI00749677	dynamin 2	1
IPI00750570	GNAS complex locus	5
IPI00758356	guanine nucleotide binding protein (G protein), beta polypeptide 2	4
IPI00856692	diacylglycerol lipase, alpha	1
IPI00876486	ectonucleotide pyrophosphatase/phosphodiesterase 7	1
IPI00885337	neuroligin 3	1
IPI00886041	neuroligin 3	1
IPI00918346	contactin 6	1
IPI01027614	dynamin 2	1
		
**OTHER**		
IPI00109727	Thy-1 cell surface antigen	5
IPI00110451	SLIT and NTRK-like family, member 1	5
IPI00115827	glioblastoma amplified sequence	5
IPI00117181	flotillin 1	3
IPI00118020	cell adhesion molecule 3	4
IPI00118075	microtubule-associated protein 2	5
IPI00119033	intercellular adhesion molecule 5, telencephalin	5
IPI00119130	BTB (POZ) domain containing 17	5
IPI00119689	adaptor-related protein complex 2, beta 1 subunit	5
IPI00119870	catenin (cadherin-associated protein), alpha 2	4
IPI00119970	contactin 2 (axonal)	5
IPI00120302	leucine-rich, glioma inactivated 1	5
IPI00120793	prion protein	4
IPI00120943	cyclin and CBS domain divalent metal cation transport mediator 1	2
IPI00121378	activated leukocyte cell adhesion molecule	5
IPI00122971	neural cell adhesion molecule 1	5
IPI00128022	protocadherin 7	2
IPI00131376	spectrin, beta, erythrocytic	2
IPI00134200	leucine rich repeat and Ig domain containing 1	5
IPI00134492	synapsin II	5
IPI00136135	catenin (cadherin-associated protein), delta 2	4
IPI00137331	CAP, adenylate cyclase-associated protein 1 (yeast)	1
IPI00153840	cell adhesion molecule 4	5
IPI00221540	ER lipid raft associated 2	1
IPI00227126	tenascin R	5
IPI00227235	ankyrin 2, brain	4
IPI00228617	guanine nucleotide binding protein (G protein), alpha inhibiting activity polypeptide 2	5
IPI00228680	neurexin III	1
IPI00229299	erythrocyte membrane protein band 4.1-like 3	4
IPI00229475	junction plakoglobin	2
IPI00229703	vesicle-associated membrane protein 2 (synaptobrevin 2)	5
IPI00230151	myelin associated glycoprotein	5
IPI00230408	microtubule-associated protein tau	2
IPI00263013	proteolipid protein 1	2
IPI00274767	glycoprotein M6B	3
IPI00310916	CD81 molecule	3
IPI00311405	poliovirus receptor-related 1 (herpesvirus entry mediator C)	5
IPI00319830	spectrin, beta, non-erythrocytic 1	5
IPI00322617	neural cell adhesion molecule 2	5
IPI00323800	neurofilament, medium polypeptide	3
IPI00329927	neurofascin	4
IPI00331579	synaptogyrin 3	5
IPI00338983	contactin associated protein 1	5
IPI00400180	amphiphysin	4
IPI00405736	CD81 molecule	1
IPI00410985	cell adhesion molecule 1	3
IPI00420467	poliovirus receptor-related 1 (herpesvirus entry mediator C)	5
IPI00420554	contactin associated protein-like 2	5
IPI00458574	cadherin 13	5
IPI00461199	bassoon presynaptic cytomatrix protein	5
IPI00467747	neuronal growth regulator 1	5
IPI00471176	hepatic and glial cell adhesion molecule	5
IPI00474209	synaptosomal-associated protein, 91 kDa	5
IPI00620207	dematin actin binding protein	1
IPI00648658	clathrin, light chain A	3
IPI00649966	synaptosomal-associated protein, 47 kDa	2
IPI00652675	limbic system-associated membrane protein	5
IPI00652902	guanine nucleotide binding protein (G protein), alpha inhibiting activity polypeptide 2	5
IPI00656204	NCK-associated protein 1	5
IPI00663736	synaptic Ras GTPase activating protein 1	3
IPI00670856	cadherin 10, type 2 (T2-cadherin)	1
IPI00675985	potassium channel tetramerization domain containing 16	5
IPI00719927	protocadherin 1	4
IPI00751569	DnaJ (Hsp40) homolog, subfamily C, member 5	5
IPI00753793	spectrin, alpha, non-erythrocytic 1	5
IPI00756921	tetraspanin 7	5
IPI00757097	SH3 and multiple ankyrin repeat domains 2	4
IPI00757771	neuroplastin	5
IPI00830223	tropomyosin 1, alpha	5
IPI00831568	L1 cell adhesion molecule	5
IPI00831624	connector enhancer of kinase suppressor of Ras 2	1
IPI00848690	lymphocyte antigen 6 complex, locus H	4
IPI00850833	cell adhesion molecule 2	4
IPI00854028	contactin associated protein 1	5
IPI00855176	protocadherin 9	4
IPI00857329	neurotrimin	5
IPI00858209	LanC lantibiotic synthetase component C-like 2 (bacterial)	5
IPI00869430	CAP, adenylate cyclase-associated protein 1 (yeast)	1
IPI00882293	membrane bound O-acyltransferase domain containing 7	3
IPI00882316	CAP, adenylate cyclase-associated protein, 2 (yeast)	5
IPI00894724	microtubule-associated protein 2	5
IPI00918899	syntaxin binding protein 5 (tomosyn)	1
IPI00929916	tenascin R	5
IPI00990801	regulating synaptic membrane exocytosis 1	2
IPI01027487	immunoglobulin superfamily, member 8	5
IPI00112226	angiopoietin-like 1	1
IPI00118420	stimulated by retinoic acid 6	1
IPI00121091	proteolipid protein 1	1
IPI00121627	cleft lip and palate associated transmembrane protein 1	2
IPI00122032	receptor accessory protein 2	1
IPI00131762	cyclin and CBS domain divalent metal cation transport mediator 2	1
IPI00131896	mitochondrial pyruvate carrier 2	1
IPI00136021	regulating synaptic membrane exocytosis 1	3
IPI00154057	protocadherin 1	1
IPI00173032	integrin, alpha E (antigen CD103, human mucosal lymphocyte antigen 1; alpha polypeptide)	1
IPI00173248	ankyrin 3, node of Ranvier (ankyrin G)	1
IPI00222908	fibronectin leucine rich transmembrane protein 2	1
IPI00228632	catenin (cadherin-associated protein), delta 2	1
IPI00230610	proteolipid protein 1	2
IPI00230751	catenin (cadherin-associated protein), alpha 2	1
IPI00273822	lysosomal-associated membrane protein 3	1
IPI00309419	leucine rich repeat containing 4C	3
IPI00313492	leucine rich repeat transmembrane neuronal 2	2
IPI00321348	immunoglobulin superfamily, member 8	2
IPI00330250	regulating synaptic membrane exocytosis 3	2
IPI00336313	protein phosphatase 1, regulatory subunit 9A	4
IPI00338880	neuronal cell adhesion molecule	2
IPI00346482	cadherin 10, type 2 (T2-cadherin)	1
IPI00351827	SH3 and multiple ankyrin repeat domains 3	3
IPI00380242	desmoglein 4	1
IPI00381088	unc-13 homolog A (C. elegans)	5
IPI00405986	erythrocyte membrane protein band 4.1-like 1	1
IPI00420570	neurofascin	1
IPI00460715	neurexin III	4
IPI00461212	oxysterol binding protein-like 8	1
IPI00466076	sidekick cell adhesion molecule 1	1
IPI00468202	trophoblast glycoprotein	3
IPI00473188	annexin A8-like 1	1
IPI00473968	cadherin 10, type 2 (T2-cadherin)	3
IPI00648543	Ras association (RalGDS/AF-6) and pleckstrin homology domains 1	1
IPI00648759	stomatin (EPB72)-like 2	5
IPI00649994	CAP, adenylate cyclase-associated protein 1 (yeast)	2
IPI00653438	trophoblast glycoprotein	2
IPI00653674	KRIT1, ankyrin repeat containing	1
IPI00670114	Ca++-dependent secretion activator	1
IPI00751974	syntrophin, alpha 1	1
IPI00756961	netrin G2	1
IPI00762484	Down syndrome cell adhesion molecule like 1	1
IPI00830145	sema domain, immunoglobulin domain (Ig), transmembrane domain (TM) and short cytoplasmic domain, (semaphorin) 4A	2
IPI00831210	neurofilament, medium polypeptide	2
IPI00831427	synovial sarcoma, X breakpoint 2 interacting protein	1
IPI00831714	leucine rich repeat containing 7	4
IPI00849429	cell adhesion molecule 1	2
IPI00853863	FERM, RhoGEF (ARHGEF) and pleckstrin domain protein 1 (chondrocyte-derived)	2
IPI00856771	podocalyxin-like 2	1
IPI00867858	protocadherin-related 15	3
IPI00876257	clathrin, light chain A	1
IPI00880812	LanC lantibiotic synthetase component C-like 1 (bacterial)	4
IPI00889283	SH3 and multiple ankyrin repeat domains 2	1
IPI00889292	FAT atypical cadherin 3	1
IPI00921638	protein tyrosine phosphatase, receptor type, f polypeptide (PTPRF), interacting protein (liprin), alpha 4	1
IPI00921642	ankyrin 2, brain	1
IPI00928058	FCH domain only 1	1
IPI00928139	DAB2 interacting protein	1
IPI00955069	contactin 5	1
IPI00987809	spectrin, beta, erythrocytic	1
IPI00988904	fibronectin leucine rich transmembrane protein 2	2
IPI00989004	cyclin and CBS domain divalent metal cation transport mediator 1	2
IPI00990422	synaptic Ras GTPase activating protein 1	4
IPI01008331	desmocollin 3	1
IPI01008664	receptor accessory protein 2	2
IPI01027584	flotillin 1	1

**Table 3:** Summary of ReViGO treemap showing the specific enrichment of proteins within the PMP enriched fraction.

	**Cluster representative**	**%**
**GO Term**		
GO:0031224	intrinsic component of membrane	16
GO:0016021	integral component of membrane	12
GO:0005886	plasma membrane	6
GO:0044459	plasma membrane part	4
GO:0034702	ion channel complex	1
GO:0031225	anchored component of membrane	1
GO:0031226	intrinsic component of plasma membrane	1
GO:0005887	integral component of plasma membrane	1
GO:0005832	chaperonin-containing T-complex	
GO:0070469	respiratory chain	
GO:0033178	proton transporting two-sector ATPase complex, catalytic domain	
GO:0031090	organelle membrane	4
GO:0005740	mitochondrial envelope	4
GO:0044429	mitochondrial part	3
GO:0042470	melanosome	2
GO:0048770	pigment granule	2
GO:0016023	cytoplasmic membrane-bounded vesicle	3
GO:0031982	vesicle	1
GO:0031410	cytoplasmic vesicle	1
GO:0045202	synapse	9
GO:0044456	synapse part	7
GO:0045111	intermediate filament cytoskeleton	3
GO:0005882	intermediate filament	3
GO:0044430	cytoskeletal part	
GO:0043005	neuron projection	4
GO:0043197	dendritic spine	1
GO:0030054	cell junction	4
GO:0031975	envelope	3

**Table 4:** Summary of ReViGO treemap showing the specific enrichment of proteins within the wash-through fraction.

	**Cluster representative**	**%**
**GO Term**		
GO:0005739	mitochondrion	21
GO:0044429	mitochondrial part	12
GO:0031967	organelle envelope	13
GO:0031090	organelle membrane	11
GO:0005829	cytosol	7
GO:0031982	vesicle	2
GO:0031410	cytoplasmic vesicle	2
GO:0016023	cytoplasmic membrane-bounded vesicle	2
GO:0043228	non-membrane-bounded organelle	2
GO:0043232	intracellular non-membrane-bounded organelle	2
GO:0005759	mitochondrial matrix	1
GO:0015630	microtubule cytoskeleton	1
GO:0070013	intracellular organelle lumen	1
GO:0005875	microtubule associated complex	1
GO:0005768	endosome	1
GO:0048770	pigment granule	1
GO:0042470	melanosome	1
GO:0005874	microtubule	
GO:0005694	chromosome	
GO:0044445	cytosolic part	
GO:0005840	ribosome	
GO:0031975	envelope	13
GO:0070469	respiratory chain	2
GO:0019898	extrinsic component of membrane	1
GO:0016469	proton-transporting two-sector ATPase complex	1
GO:0009898	cytoplasmic side of plasma membrane	1
GO:0030529	ribonucleoprotein complex	
GO:0000502	proteasome complex	
GO:0044448	cell cortex part	
GO:0031974	membrane-enclosed lumen	1
GO:0012505	endomembrane system	1
GO:0045177	apical part of cell	
GO:0000267	cell fraction	
