# Supplementary material for: An effective plasma membrane proteomics approach for small tissue samples
Source: Sci Rep. 2015 Jun 5;5:10917. doi: 10.1038/srep10917 (PMC4456939; doi:10.1038/srep10917)
Supplement: Supplementary Information [file srep10917-s1.pdf]

**An effective plasma membrane proteomics approach for small tissue samples**

Katrien Smolders, Nathalie Lombaert, Dirk Valkenborg, Geert Baggerman, Lutgarde Arckens

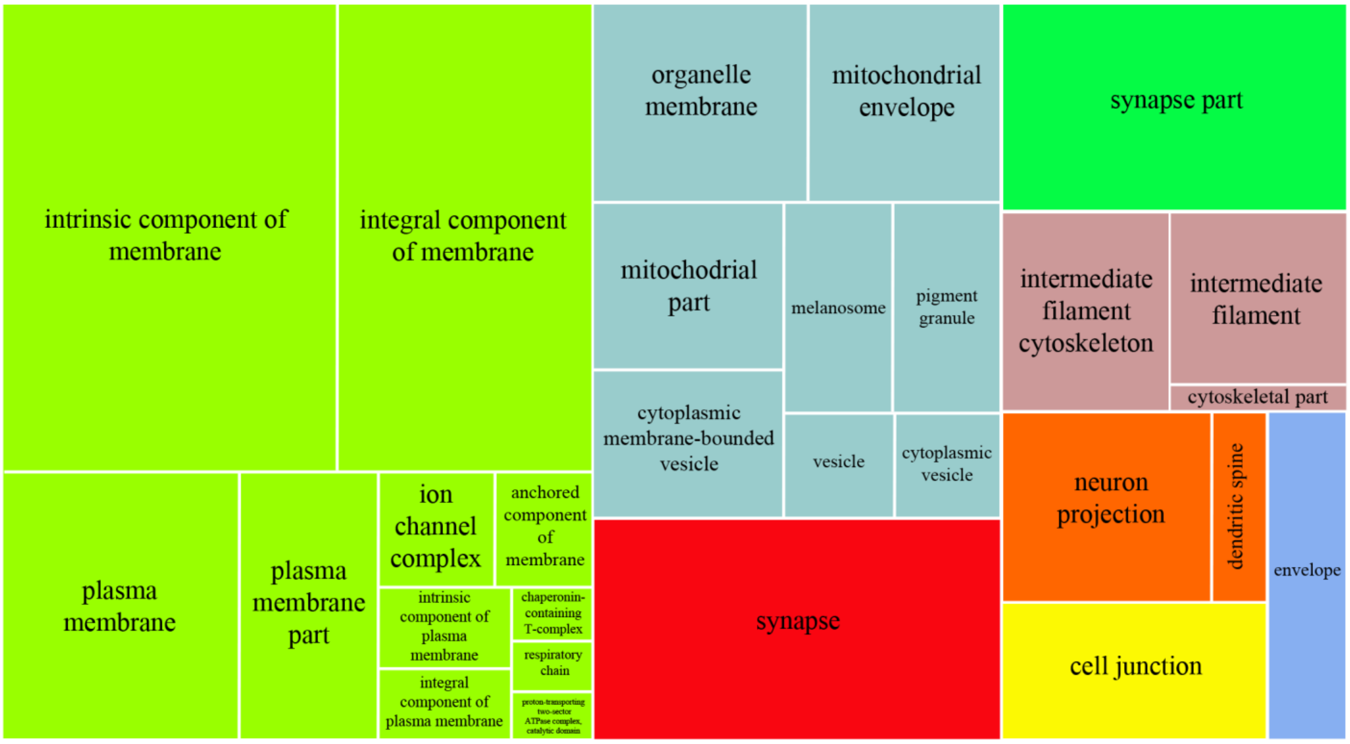


**Supplementary Figure 1** Selective enrichment of plasma membrane proteins in the plasma membrane protein enriched fraction. Treemap adapted from ReViGO shows the specific enrichment of all proteins within the plasma membrane protein (PMP) enriched fraction relative to a background that was built by merging all proteins identified in the PMP enriched with those from the wash-through fraction. Each rectangle is a single cluster representative and its size is in proportion to ReViGO derived p-values based on analysis of cellular component ontology enrichment in the DAVID web tool. The different colours represent 'superclusters' comprising related cluster representatives.

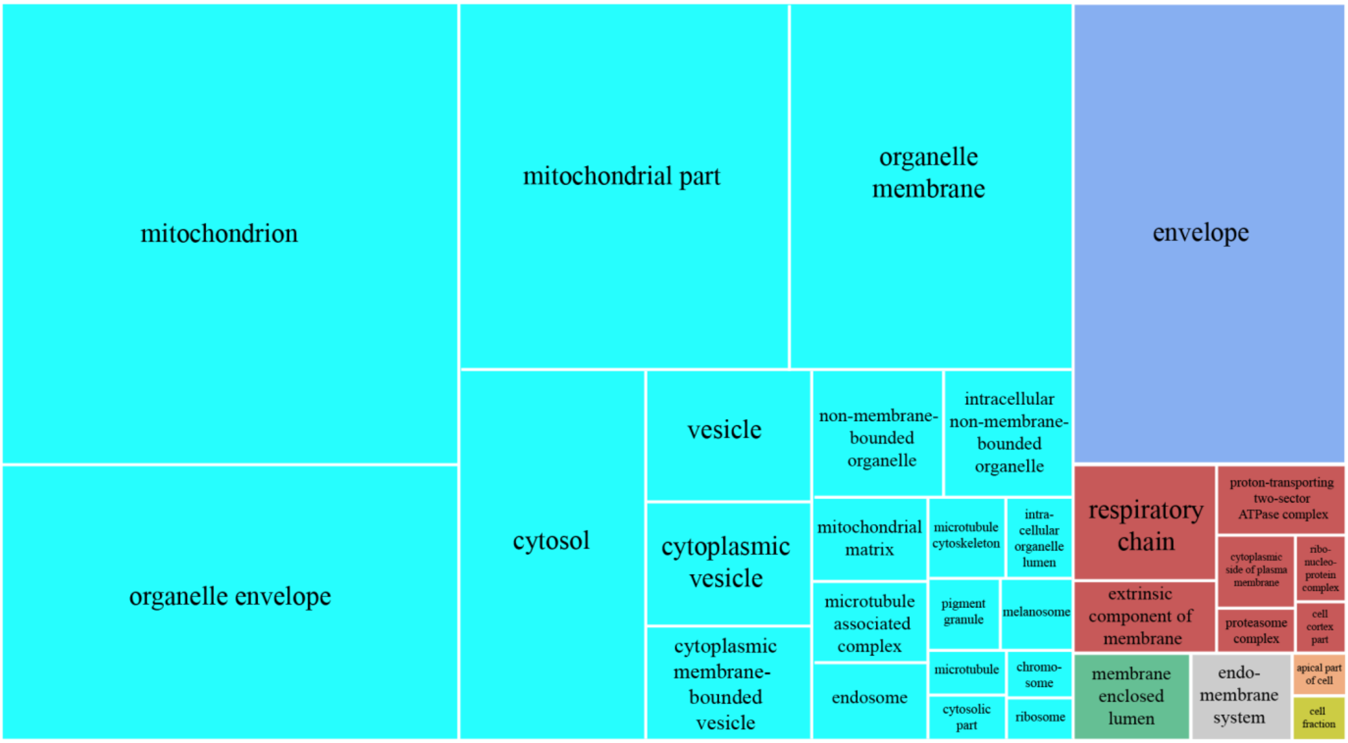


**Supplementary Figure 2** Selective enrichment of mitochondrial proteins in the wash-through fraction. Treemap adapted from ReViGO shows the specific enrichment of all proteins within the wash-through fraction relative to a background that was built by merging all proteins identified in the PMP enriched with those from the wash-through fraction. Each rectangle is a single cluster representative and its size is in proportion to ReViGO derived p-values based on analysis of cellular component ontology enrichment in the DAVID web tool. Different colours represent different 'superclusters', comprising related cluster representatives.

## Supplementary Table 1 372 extra PMPs in the PMP enriched fraction (2° annotations in IPA, DAVID and with literature search[6])

| Accession no. | Protein name | in no. of samples |
|---|---|---|
| **Ion channel** | | |
| IPI00122547 | voltage-dependent anion channel 2 | 2 |
| IPI00230540 | voltage-dependent anion channel 1 | 5 |
| IPI00876341 | voltage-dependent anion channel 3 | 4 |
| IPI00230023 | inositol 1,4,5-trisphosphate receptor, type 1 | 5 |
| IPI00750490 | voltage-dependent anion channel 2 | 2 |
| IPI00857120 | inositol 1,4,5-trisphosphate receptor, type 2 | 1 |
| **Transporter** | | |
| IPI00111181 | vacuolar protein sorting 35 homolog (S. cerevisiae) | 5 |
| IPI00115564 | solute carrier family 25 (mitochondrial carrier; adenine nucleotide translocator), member 4 | 5 |
| IPI00118930 | N-ethylmaleimide-sensitive factor attachment protein, alpha | 5 |
| IPI00118986 | ATP synthase, H+ transporting, mitochondrial F1 complex, O subunit | 5 |
| IPI00119113 | ATPase, H+ transporting, lysosomal 56/58kDa, V1 subunit B2 | 5 |
| IPI00119115 | ATPase, H+ transporting, lysosomal 31kDa, V1 subunit E1 | 5 |
| IPI00124771 | solute carrier family 25 (mitochondrial carrier; phosphate carrier), member 3 | 5 |
| IPI00127841 | solute carrier family 25 (mitochondrial carrier; adenine nucleotide translocator), member 5 | 5 |
| IPI00129618 | synaptotagmin I | 5 |
| IPI00129622 | synaptotagmin II | 2 |
| IPI00130186 | ATPase, H+ transporting, lysosomal 42kDa, V1 subunit C1 | 5 |
| IPI00130280 | ATP synthase, H+ transporting, mitochondrial F1 complex, alpha subunit 1, cardiac muscle | 5 |
| IPI00170310 | solute carrier family 8 (sodium/calcium exchanger), member 2 | 5 |
| IPI00228525 | ATPase, H+ transporting, lysosomal V0 subunit a1 | 4 |
| IPI00228526 | ATPase, H+ transporting, lysosomal V0 subunit a1 | 4 |
| IPI00310131 | adaptor-related protein complex 2, alpha 2 subunit | 5 |
| IPI00311461 | ATPase, H+ transporting, lysosomal 50/57kDa, V1 subunit H | 5 |
| IPI00313841 | ATPase, H+ transporting, lysosomal 38kDa, V0 subunit d1 | 5 |
| IPI00341282 | ATP synthase, H+ transporting, mitochondrial Fo complex, subunit B1 | 5 |
| IPI00415402 | syntaxin binding protein 1 | 5 |
| IPI00420426 | adaptor-related protein complex 3, beta 2 subunit | 5 |
| IPI00465810 | synaptic vesicle glycoprotein 2A | 5 |
| IPI00468481 | ATP synthase, H+ transporting, mitochondrial F1 complex, beta polypeptide | 5 |
| IPI00468900 | ATPase, Ca++ transporting, cardiac muscle, slow twitch 2 | 5 |
| IPI00553576 | ATP-binding cassette, sub-family D (ALD), member 3 | 4 |
| IPI00622811 | adaptor-related protein complex 2, mu 1 subunit | 5 |
| IPI00622911 | adaptor-related protein complex 2, alpha 1 subunit | 5 |
| IPI00656325 | N-ethylmaleimide-sensitive factor | 5 |
| IPI00750074 | ATP synthase, H+ transporting, mitochondrial F1 complex, gamma polypeptide 1 | 5 |
| IPI00751137 | translocase of outer mitochondrial membrane 70 homolog A (S. cerevisiae) | 2 |
| IPI00753468 | adaptor-related protein complex 2, alpha 2 subunit | 5 |
| IPI00885385 | syntaxin 1A (brain) | 5 |
| IPI00120503 | coatomer protein complex, subunit beta 1 | 5 |
| IPI00648357 | solute carrier family 25 (mitochondrial carrier; oxoglutarate carrier), member 11 | 3 |

| | | |
|---|---|---|
| IPI00655041 | exocyst complex component 2 | 4 |
| IPI00775883 | synaptophysin | 1 |
| IPI00896643 | coatomer protein complex, subunit gamma 2 | 1 |
| IPI00169896 | solute carrier family 44 (choline transporter), member 2 | 5 |
| IPI00649186 | solute carrier family 44 (choline transporter), member 2 | 5 |
| IPI00124286 | adaptor-related protein complex 3, mu 2 subunit | 3 |
| IPI00127867 | synaptotagmin V | 3 |
| IPI00230754 | solute carrier family 25 (mitochondrial carrier; oxoglutarate carrier), member 11 | 2 |
| IPI00315135 | translocase of outer mitochondrial membrane 22 homolog (yeast) | 5 |
| IPI00112776 | ATPase, aminophospholipid transporter (APLT), class I, type 8A, member 1 | 3 |
| IPI00123505 | synaptophysin | 1 |
| IPI00127871 | synaptotagmin VII | 1 |
| IPI00135048 | vacuolar protein sorting 33 homolog A (S. cerevisiae) | 1 |
| IPI00387189 | vacuolar protein sorting 18 homolog (S. cerevisiae) | 1 |
| IPI00468100 | piccolo presynaptic cytomatrix protein | 1 |
| IPI00828526 | ATPase, Cu++ transporting, beta polypeptide | 1 |
| IPI00857093 | amyloid beta (A4) precursor protein-binding, family A, member 2 | 1 |
| IPI00986291 | amyloid beta (A4) precursor protein-binding, family A, member 1 | 1 |
| IPI00986992 | ATPase, H+ transporting, lysosomal V0 subunit a1 | 1 |
| **Kinase** | | |
| IPI00115875 | phosphatidylinositol 4-kinase, catalytic, alpha | 5 |
| IPI00122069 | protein kinase C, gamma | 5 |
| IPI00123613 | protein kinase C and casein kinase substrate in neurons 1 | 2 |
| IPI00136703 | creatine kinase, brain | 5 |
| IPI00169788 | protein kinase, cAMP-dependent, regulatory, type II, alpha | 2 |
| IPI00224570 | protein kinase, cAMP-dependent, regulatory, type II, beta | 5 |
| IPI00228045 | calcium/calmodulin-dependent protein kinase II gamma | 5 |
| IPI00356608 | AP2 associated kinase 1 | 5 |
| IPI00604947 | trio Rho guanine nucleotide exchange factor | 4 |
| IPI00621806 | calcium/calmodulin-dependent protein kinase II alpha | 5 |
| IPI00649586 | NME/NM23 nucleoside diphosphate kinase 1 | 5 |
| IPI00757755 | protein kinase C, beta | 3 |
| IPI00845840 | pyruvate kinase, muscle | 5 |
| IPI00928559 | hexokinase 1 | 5 |
| IPI00649373 | G protein-coupled receptor kinase interacting ArfGAP 1 | 5 |
| IPI00122486 | CaM kinase-like vesicle-associated | 5 |
| IPI00761729 | doublecortin-like kinase 1 | 5 |
| IPI00321446 | protein kinase C, alpha | 3 |
| IPI00336318 | phosphatidylinositol-5-phosphate 4-kinase, type II, beta | 4 |
| IPI00624499 | calcium/calmodulin-dependent protein kinase II beta | 5 |
| IPI00756265 | citron rho-interacting serine/threonine kinase | 1 |
| IPI00762727 | MAP/microtubule affinity-regulating kinase 2 | 3 |
| IPI00858128 | calcium/calmodulin-dependent protein kinase II delta | 4 |
| IPI00974715 | citron rho-interacting serine/threonine kinase | 1 |
| IPI00108150 | Rho-associated, coiled-coil containing protein kinase 2 | 2 |
| IPI00227898 | protein kinase C, beta | 1 |

| | | |
|---|---|---|
| IPI00474243 | mitogen-activated protein kinase 1 | 4 |
| IPI00857865 | calcium/calmodulin-dependent protein kinase II delta | 1 |
| IPI00116546 | protein kinase, cAMP-dependent, regulatory, type II, alpha | 1 |
| IPI00128363 | MAP/microtubule affinity-regulating kinase 1 | 1 |
| IPI00227900 | protein kinase, cAMP-dependent, catalytic, alpha | 1 |
| IPI00261299 | protein kinase, cAMP-dependent, regulatory, type II, alpha | 2 |
| IPI00330332 | diacylglycerol kinase, zeta | 3 |
| IPI00395144 | death-associated protein kinase 1 | 1 |
| IPI00475262 | phosphatidylinositol-4-phosphate 5-kinase, type I, alpha | 1 |
| IPI00626695 | obscurin, cytoskeletal calmodulin and titin-interacting RhoGEF | 1 |
| IPI00830616 | protein tyrosine kinase 2 beta | 4 |
| IPI00943997 | trio Rho guanine nucleotide exchange factor | 4 |
| IPI00268673 | mechanistic target of rapamycin (serine/threonine kinase) | 3 |
| IPI00929894 | mechanistic target of rapamycin (serine/threonine kinase) | 3 |
| **Peptidase** | | |
| IPI00119876 | dynein, cytoplasmic 1, heavy chain 1 | 5 |
| IPI00230108 | protein disulfide isomerase family A, member 3 | 5 |
| IPI00608097 | aminopeptidase puromycin sensitive | 5 |
| IPI00396687 | dipeptidyl-peptidase 10 (non-functional) | 5 |
| **Phosphatase** | | |
| IPI00123862 | protein phosphatase 1, catalytic subunit, gamma isoform | 5 |
| IPI00310091 | protein phosphatase 2, regulatory subunit A, alpha | 5 |
| IPI00756703 | protein phosphatase 3, catalytic subunit, alpha isozyme | 5 |
| IPI00881540 | synaptojanin 1 | 5 |
| IPI00110990 | dual specificity phosphatase 3 | 1 |
| IPI00134135 | acid phosphatase 1, soluble | 1 |
| IPI00131028 | myotubularin related protein 1 | 1 |
| IPI00875081 | myotubularin related protein 1 | 1 |
| **Transcription regulator** | | |
| IPI00227392 | tyrosine 3-monooxygenase/tryptophan 5-monooxygenase activation protein, eta | 4 |
| IPI00321718 | prohibitin 2 | 5 |
| IPI00623970 | huntingtin | 5 |
| IPI00125899 | catenin (cadherin-associated protein), beta 1, 88kDa | 5 |
| IPI00133440 | prohibitin | 5 |
| IPI00462789 | proteasome (prosome, macropain) 26S subunit, ATPase, 5 | 3 |
| IPI00515155 | nucleophosmin (nucleolar phosphoprotein B23, numatrin) | 2 |
| IPI00625369 | ubinuclein 1 | 1 |
| **Translation regulator** | | |
| IPI00119667 | eukaryotic translation elongation factor 1 alpha 2 | 1 |
| IPI00123604 | ribosomal protein SA | 5 |
| IPI00307837 | eukaryotic translation elongation factor 1 alpha 1 | 5 |
| IPI00331552 | poly(A) binding protein, cytoplasmic 1 | 2 |
| **Growth factor** | | |
| IPI00454159 | chondroitin sulfate proteoglycan 5 (neuroglycan C) | 2 |
| IPI00114341 | Norrie disease (pseudoglioma) | 2 |
| IPI00652233 | chondroitin sulfate proteoglycan 5 (neuroglycan C) | 2 |

| | | |
|---|---|---|
| **Enzyme** | | |
| IPI00113112 | RAB3B, member RAS oncogene family | 5 |
| IPI00113141 | citrate synthase | 5 |
| IPI00113223 | fatty acid synthase | 5 |
| IPI00114375 | dihydropyrimidinase-like 2 | 5 |
| IPI00115824 | nipsnap homolog 1 (C. elegans) | 5 |
| IPI00116074 | aconitase 2, mitochondrial | 5 |
| IPI00116498 | tyrosine 3-monooxygenase/tryptophan 5-monooxygenase activation protein, zeta | 4 |
| IPI00117312 | glutamic-oxaloacetic transaminase 2, mitochondrial | 5 |
| IPI00119138 | ubiquinol-cytochrome c reductase core protein II | 5 |
| IPI00120719 | cytochrome c oxidase subunit Va | 5 |
| IPI00122965 | RAB3A, member RAS oncogene family | 5 |
| IPI00130118 | RAB10, member RAS oncogene family | 4 |
| IPI00130353 | valyl-tRNA synthetase | 5 |
| IPI00132002 | microsomal glutathione S-transferase 3 | 5 |
| IPI00132042 | pyruvate dehydrogenase (lipoamide) beta | 5 |
| IPI00134809 | dihydrolipoamide S-succinyltransferase (E2 component of 2-oxo-glutarate complex) | 4 |
| IPI00137227 | RAB2A, member RAS oncogene family | 3 |
| IPI00138406 | RAP1A, member of RAS oncogene family | 5 |
| IPI00153660 | dihydrolipoamide S-acetyltransferase | 5 |
| IPI00154054 | acetyl-CoA acetyltransferase 1 | 5 |
| IPI00221402 | aldolase A, fructose-bisphosphate | 1 |
| IPI00221754 | atlastin GTPase 1 | 5 |
| IPI00222496 | protein disulfide isomerase family A, member 6 | 5 |
| IPI00223092 | hydroxyacyl-CoA dehydrogenase/3-ketoacyl-CoA thiolase/enoyl-CoA hydratase (trifunctional protein), alpha subunit | 1 |
| IPI00224518 | RAB5C, member RAS oncogene family | 5 |
| IPI00229080 | heat shock protein 90kDa alpha (cytosolic), class B member 1 | 5 |
| IPI00229598 | 2',3'-cyclic nucleotide 3' phosphodiesterase | 5 |
| IPI00229796 | phosphorylase, glycogen; brain | 5 |
| IPI00230351 | succinate dehydrogenase complex, subunit A, flavoprotein (Fp) | 5 |
| IPI00265467 | regulator of G-protein signaling 6 | 2 |
| IPI00308885 | heat shock 60kDa protein 1 (chaperonin) | 5 |
| IPI00318496 | glutamate decarboxylase 1 (brain, 67kDa) | 3 |
| IPI00318522 | glutamate decarboxylase 2 (pancreatic islets and brain, 65kDa) | 2 |
| IPI00319992 | heat shock 70kDa protein 5 (glucose-regulated protein, 78kDa) | 5 |
| IPI00323357 | heat shock 70kDa protein 8 | 2 |
| IPI00323592 | malate dehydrogenase 2, NAD (mitochondrial) | 5 |
| IPI00330804 | heat shock protein 90kDa alpha (cytosolic), class A member 1 | 5 |
| IPI00337893 | pyruvate dehydrogenase (lipoamide) alpha 1 | 5 |
| IPI00339916 | glutamyl-prolyl-tRNA synthetase | 1 |
| IPI00406442 | succinate-CoA ligase, alpha subunit | 5 |
| IPI00408892 | RAB7A, member RAS oncogene family | 5 |
| IPI00465648 | dynamin 1 | 5 |
| IPI00467833 | triosephosphate isomerase 1 | 2 |
| IPI00553820 | acyl-CoA synthetase long-chain family member 6 | 4 |
| IPI00625738 | dynamin 3 | 3 |

| | | |
|---|---|---|
| IPI00648105 | peroxiredoxin 1 | 5 |
| IPI00649647 | ras-related C3 botulinum toxin substrate 3 (rho family, small GTP binding protein Rac3) | 1 |
| IPI00653598 | ubiquinol-cytochrome c reductase core protein I | 5 |
| IPI00719841 | oxoglutarate (alpha-ketoglutarate) dehydrogenase (lipoamide) | 5 |
| IPI00754398 | lactate dehydrogenase A | 3 |
| IPI00755031 | glutaminase | 4 |
| IPI00762047 | ATP citrate lyase | 5 |
| IPI00845528 | O-linked N-acetylglucosamine (GlcNAc) transferase | 5 |
| IPI00874456 | dihydrolipoamide dehydrogenase | 5 |
| IPI00930784 | NADH dehydrogenase (ubiquinone) Fe-S protein 1, 75kDa (NADH-coenzyme Q reductase) | 5 |
| IPI00470004 | endonuclease domain containing 1 | 2 |
| IPI00115089 | ectonucleoside triphosphate diphosphohydrolase 2 | 5 |
| IPI00116770 | RAB18, member RAS oncogene family | 4 |
| IPI00122684 | enolase 2 (gamma, neuronal) | 5 |
| IPI00130489 | RAB35, member RAS oncogene family | 2 |
| IPI00132410 | RAB5A, member RAS oncogene family | 5 |
| IPI00134941 | Kirsten rat sarcoma viral oncogene homolog | 1 |
| IPI00162949 | CDP-diacylglycerol--inositol 3-phosphatidyltransferase | 5 |
| IPI00221613 | ADP-ribosylation factor 1 | 5 |
| IPI00307931 | ras homolog family member T1 | 4 |
| IPI00458976 | RAB5B, member RAS oncogene family | 3 |
| IPI00649532 | regulator of G-protein signaling 7 | 5 |
| IPI00757464 | RAB14, member RAS oncogene family | 2 |
| IPI00121575 | ALG2, alpha-1,3/1,6-mannosyltransferase | 2 |
| IPI00123181 | myosin, heavy chain 9, non-muscle | 5 |
| IPI00126548 | 1-acylglycerol-3-phosphate O-acyltransferase 1 | 3 |
| IPI00230035 | DEAD (Asp-Glu-Ala-Asp) box helicase 3, X-linked | 2 |
| IPI00337980 | RAB21, member RAS oncogene family | 3 |
| IPI00653283 | guanylate cyclase 1, soluble, beta 3 | 4 |
| IPI00845769 | glycerol-3-phosphate dehydrogenase 1-like | 1 |
| IPI00845858 | dihydrolipoamide S-succinyltransferase (E2 component of 2-oxo-glutarate complex) | 1 |
| IPI00923037 | ubiquitin C | 2 |
| IPI00113638 | junctophilin 2 | 1 |
| IPI00117705 | dolichyl-diphosphooligosaccharide--protein glycosyltransferase subunit (non-catalytic) | 5 |
| IPI00125850 | RASD family, member 2 | 1 |
| IPI00229613 | microtubule-actin crosslinking factor 1 | 1 |
| IPI00309035 | ribophorin I | 3 |
| IPI00317740 | guanine nucleotide binding protein (G protein), beta polypeptide 2-like 1 | 3 |
| IPI00338854 | guanine nucleotide binding protein (G protein), alpha inhibiting activity polypeptide 3 | 3 |
| IPI00404182 | ras homolog family member B | 1 |
| IPI00410819 | dolichyl-diphosphooligosaccharide--protein glycosyltransferase subunit (non-catalytic) | 4 |
| IPI00453834 | acyl-CoA synthetase bubblegum family member 1 | 3 |
| IPI00466992 | WD repeat and FYVE domain containing 3 | 1 |
| IPI00474825 | sphingomyelin phosphodiesterase 2, neutral membrane (neutral sphingomyelinase) | 1 |
| IPI00553304 | arachidonate 15-lipoxygenase | 1 |
| IPI00555130 | RAB3 GTPase activating protein subunit 2 (non-catalytic) | 1 |

| | | |
|---|---|---|
| IPI00623742 | phosphodiesterase 2A, cGMP-stimulated | 2 |
| IPI00652772 | phosphodiesterase 2A, cGMP-stimulated | 1 |
| IPI00652871 | Wolfram syndrome 1 (wolframin) | 1 |
| IPI00653348 | regulator of G-protein signaling 6 | 3 |
| IPI00662806 | glutaminase | 1 |
| IPI00666323 | neural precursor cell expressed, developmentally down-regulated 4 | 2 |
| IPI00757004 | phospholipase C, gamma 1 | 2 |
| IPI00762623 | phosphodiesterase 2A, cGMP-stimulated | 1 |
| IPI00816967 | B-Raf proto-oncogene, serine/threonine kinase | 1 |
| IPI00858310 | aldolase A, fructose-bisphosphate | 4 |
| IPI00875619 | transglutaminase 3 | 1 |
| IPI00885631 | acyl-CoA synthetase bubblegum family member 1 | 1 |
| IPI00886297 | heat shock 70kDa protein 8 | 3 |
| IPI00987804 | RAB3C, member RAS oncogene family | 1 |
| IPI00988063 | triosephosphate isomerase 1 | 1 |
| IPI00990097 | phospholipase C, beta 1 (phosphoinositide-specific) | 1 |
| **Other** | | |
| IPI00330476 | cytoplasmic FMR1 interacting protein 1 | 2 |
| IPI00109073 | tubulin, beta 4A class IVa | 5 |
| IPI00112251 | tubulin, beta 3 class III | 5 |
| IPI00114241 | neurofilament, heavy polypeptide | 5 |
| IPI00116283 | chaperonin containing TCP1, subunit 3 (gamma) | 5 |
| IPI00117352 | tubulin, beta class I | 5 |
| IPI00118384 | tyrosine 3-monooxygenase/tryptophan 5-monooxygenase activation protein, epsilon | 5 |
| IPI00119024 | ADP-ribosylation factor-like 6 interacting protein 5 | 5 |
| IPI00119067 | tubulin polymerization promoting protein | 5 |
| IPI00119618 | calnexin | 3 |
| IPI00122928 | tubulin, beta 6 class V | 4 |
| IPI00130920 | microtubule-associated protein 1B | 5 |
| IPI00133903 | heat shock 70kDa protein 9 (mortalin) | 5 |
| IPI00134344 | spectrin, beta, non-erythrocytic 2 | 5 |
| IPI00135965 | internexin neuronal intermediate filament protein, alpha | 5 |
| IPI00221608 | SAMM50 sorting and assembly machinery component | 5 |
| IPI00225322 | adducin 1 (alpha) | 5 |
| IPI00230707 | tyrosine 3-monooxygenase/tryptophan 5-monooxygenase activation protein, gamma | 5 |
| IPI00323600 | coronin, actin binding protein, 1A | 2 |
| IPI00338039 | tubulin, beta 2A class IIa | 5 |
| IPI00400328 | adducin 2 (beta) | 5 |
| IPI00405459 | neuronal guanine nucleotide exchange factor | 5 |
| IPI00408909 | microtubule-associated protein 1A | 5 |
| IPI00515398 | myosin, heavy chain 10, non-muscle | 5 |
| IPI00554928 | neurofilament, light polypeptide | 5 |
| IPI00625729 | keratin 1 | 4 |
| IPI00853932 | Dmx-like 2 | 5 |
| IPI00874440 | septin 7 | 4 |
| IPI00880617 | SEC22 vesicle trafficking protein homolog B (S. cerevisiae) (gene/pseudogene) | 5 |

| | | |
|---|---|---|
| IPI00881762 | capping protein (actin filament) muscle Z-line, alpha 2 | 4 |
| IPI00886212 | oxysterol binding protein-like 1A | 5 |
| IPI00896700 | microtubule-associated protein 1B | 5 |
| IPI00125992 | neuronal pentraxin I | 4 |
| IPI00126405 | myelin oligodendrocyte glycoprotein | 5 |
| IPI00135563 | neurocan | 5 |
| IPI00347110 | keratin 73 | 3 |
| IPI00670400 | apolipoprotein O | 5 |
| IPI00869394 | brevican | 5 |
| IPI00928284 | prosaposin | 2 |
| IPI00117803 | astrotactin 1 | 5 |
| IPI00122399 | golgi glycoprotein 1 | 4 |
| IPI00129526 | heat shock protein 90kDa beta (Grp94), member 1 | 5 |
| IPI00130577 | SLIT-ROBO Rho GTPase activating protein 2 | 2 |
| IPI00130833 | FK506 binding protein 8, 38kDa | 1 |
| IPI00135475 | drebrin 1 | 4 |
| IPI00229509 | plectin | 5 |
| IPI00230068 | kinesin-associated protein 3 | 3 |
| IPI00270767 | reticulon 4 | 3 |
| IPI00323483 | programmed cell death 6 interacting protein | 5 |
| IPI00330606 | MAP-kinase activating death domain | 4 |
| IPI00353563 | fascin actin-bundling protein 1 | 4 |
| IPI00405625 | cytoplasmic FMR1 interacting protein 2 | 4 |
| IPI00415558 | synaptopodin | 4 |
| IPI00470126 | keratin 5 | 1 |
| IPI00473320 | actin, beta | 1 |
| IPI00649394 | protein phosphatase 1, regulatory subunit 9B | 4 |
| IPI00652983 | ELKS/RAB6-interacting/CAST family member 2 | 4 |
| IPI00828453 | SH3-domain GRB2-like endophilin B2 | 1 |
| IPI00828583 | Rho/Rac guanine nucleotide exchange factor (GEF) 2 | 5 |
| IPI00880561 | small ArfGAP 1 | 4 |
| IPI00918329 | clusterin | 5 |
| IPI00923036 | programmed cell death 6 interacting protein | 5 |
| IPI00929837 | hepatocyte growth factor-regulated tyrosine kinase substrate | 1 |
| IPI00989903 | actinin, alpha 1 | 1 |
| IPI01008141 | plectin | 5 |
| IPI00126759 | leucine rich repeat containing 8 family, member A | 4 |
| IPI00469999 | netrin G1 | 5 |
| IPI00918250 | versican | 1 |
| IPI00673967 | ankyrin repeat and sterile alpha motif domain containing 1B | 3 |
| IPI00130766 | proline-rich transmembrane protein 1 | 1 |
| IPI00110850 | actin, beta | 4 |
| IPI00112948 | reticulon 3 | 1 |
| IPI00116112 | dynactin 2 (p50) | 5 |
| IPI00122865 | secretory carrier membrane protein 5 | 1 |
| IPI00133706 | RAB1B, member RAS oncogene family | 1 |

| | | |
|---|---|---|
| IPI00135677 | EH-domain containing 3 | 1 |
| IPI00169647 | SH3-domain GRB2-like endophilin B2 | 2 |
| IPI00227851 | LPS-responsive vesicle trafficking, beach and anchor containing | 1 |
| IPI00354819 | myosin, light chain 6, alkali, smooth muscle and non-muscle | 1 |
| IPI00380436 | actinin, alpha 1 | 4 |
| IPI00474792 | transmembrane protein 33 | 5 |
| IPI00653749 | abl-interactor 1 | 3 |
| IPI00830860 | cytoplasmic linker associated protein 2 | 5 |
| IPI00853823 | Rap guanine nucleotide exchange factor (GEF) 2 | 1 |
| IPI00856365 | dynactin 1 | 3 |
| IPI00882228 | TRK-fused gene | 2 |
| IPI00223596 | myelin basic protein | 2 |
| IPI00880733 | tetraspanin 2 | 1 |
| IPI00752419 | talin 2 | 1 |
| IPI00120546 | PRA1 domain family, member 2 | 3 |
| IPI00108372 | Ras protein-specific guanine nucleotide-releasing factor 2 | 1 |
| IPI00116752 | SEC23 interacting protein | 1 |
| IPI00118616 | family with sequence similarity 126, member A | 1 |
| IPI00121142 | tetratricopeptide repeat domain 8 | 2 |
| IPI00123577 | uroplakin 3A | 1 |
| IPI00129907 | acyl-CoA binding domain containing 3 | 1 |
| IPI00137087 | ADP-ribosylation factor guanine nucleotide-exchange factor 2 (brefeldin A-inhibited) | 2 |
| IPI00139301 | keratin 5 | 1 |
| IPI00223400 | neurofibromin 1 | 1 |
| IPI00226564 | KIAA1524 | 1 |
| IPI00265864 | toll interacting protein | 1 |
| IPI00320831 | neurobeachin | 4 |
| IPI00330619 | Vac14 homolog (S. cerevisiae) | 1 |
| IPI00331516 | drebrin 1 | 1 |
| IPI00378580 | DnaJ (Hsp40) homolog, subfamily C, member 13 | 3 |
| IPI00380956 | abl-interactor 2 | 5 |
| IPI00420868 | RAS p21 protein activator 4 | 2 |
| IPI00460662 | seizure related 6 homolog (mouse)-like 2 | 4 |
| IPI00462397 | MAP-kinase activating death domain | 1 |
| IPI00464181 | phosphatidylinositol-3,4,5-trisphosphate-dependent Rac exchange factor 1 | 1 |
| IPI00649259 | hepatocyte growth factor-regulated tyrosine kinase substrate | 2 |
| IPI00652450 | lethal giant larvae homolog 2 (Drosophila) | 1 |
| IPI00661414 | actin related protein 2/3 complex, subunit 2, 34kDa | 1 |
| IPI00761751 | kinesin family member 13B | 1 |
| IPI00798490 | microtubule-associated protein 7 | 1 |
| IPI00817008 | regulator of G-protein signaling 7 binding protein | 2 |
| IPI00831544 | cytoplasmic FMR1 interacting protein 1 | 2 |
| IPI00850843 | AHNAK nucleoprotein 2 | 1 |
| IPI00856331 | phosphatidylethanolamine binding protein 1 | 2 |
| IPI00856554 | islet cell autoantigen 1, 69kDa | 1 |
| IPI00857756 | v-crk avian sarcoma virus CT10 oncogene homolog | 1 |

| | | |
|---|---|---|
| IPI00857866 | cortactin binding protein 2 | 1 |
| IPI00918287 | Rho GTPase activating protein 26 | 1 |
| IPI00928160 | Ras protein-specific guanine nucleotide-releasing factor 2 | 1 |
| IPI00956958 | drebrin 1 | 1 |
| IPI00988089 | FYVE, RhoGEF and PH domain containing 5 | 2 |
| IPI01023232 | microtubule-associated protein 4 | 1 |
| IPI00122312 | fibrinogen gamma chain | 1 |
| IPI00170121 | leucine-rich repeat LGI family, member 3 | 3 |
| IPI00223378 | myelin basic protein | 3 |
| IPI00223699 | Fraser extracellular matrix complex subunit 1 | 1 |
| IPI00469172 | versican | 5 |
| IPI00420385 | septin 11 | 1 |
| IPI00553798 | AHNAK nucleoprotein | 1 |
| IPI00752631 | catenin (cadherin-associated protein), delta 1 | 1 |
| IPI00132812 | proline rich 7 (synaptic) | 4 |
| IPI00757551 | Na+/K+ transporting ATPase interacting 4 | 1 |
| IPI00876467 | Na+/K+ transporting ATPase interacting 4 | 1 |
| IPI00881786 | potassium channel tetramerization domain containing 19 | 1 |

## Supplementary Table 2 222 extra cell surface-associated proteins in the PMP enriched fraction (2° annotations in IPA, DAVID and with literature search[6])

| | Accession no. | Protein name | in no. of samples |
|---|---|---|---|
| **Cell surface** | **Transporter** | | |
| | IPI00118832 | endoplasmic reticulum protein 29 | 1 |
| | **Peptidase** | | |
| | IPI00130000 | aminopeptidase puromycin sensitive | 5 |
| | IPI00473685 | ubiquitin specific peptidase 14 (tRNA-guanine transglycosylase) | 1 |
| | IPI00885558 | protein disulfide isomerase family A, member 3 | 2 |
| | **Enzyme** | | |
| | IPI00554929 | heat shock protein 90kDa alpha (cytosolic), class B member 1 | 5 |
| | IPI00118158 | mutS homolog 2 | 1 |
| | **Other** | | |
| | IPI00331556 | heat shock 70kDa protein 4 | 4 |
| | IPI00110827 | actin, alpha 1, skeletal muscle | 1 |
| **Cellular membrane** | **Ion channel** | | |
| | IPI00122548 | voltage-dependent anion channel 3 | 4 |
| | IPI00416130 | peroxisomal biogenesis factor 5-like | 1 |
| | **Transporter** | | |
| | IPI01023167 | translocase of outer mitochondrial membrane 70 homolog A (S. cerevisiae) | 2 |
| | IPI00135646 | ATP-binding cassette, sub-family D (ALD), member 3 | 5 |
| | IPI00112414 | CSE1 chromosome segregation 1-like (yeast) | 5 |
| | IPI00118787 | ATPase, H+ transporting, lysosomal 34kDa, V1 subunit D | 5 |

| | | |
|---|---|---|
| IPI00128071 | USO1 vesicle transport factor | 3 |
| IPI00136246 | phosphatidylinositol transfer protein, membrane-associated 1 | 1 |
| IPI00377728 | translocase of outer mitochondrial membrane 70 homolog A (S. cerevisiae) | 1 |
| IPI00403807 | solute carrier family 9, subfamily A (NHE8, cation proton antiporter 8), member 8 | 1 |
| IPI00659170 | USO1 vesicle transport factor | 2 |
| IPI00742406 | ATPase, class II, type 9B | 1 |
| IPI00751770 | PITPNM family member 3 | 1 |
| IPI00751937 | ATPase, class II, type 9A | 1 |
| IPI00785352 | ATPase, aminophospholipid transporter, class I, type 8B, member 3 | 1 |
| IPI00856425 | ATP-binding cassette, sub-family B (MDR/TAP), member 8 | 1 |
| IPI00880276 | ATPase, H+ transporting, lysosomal accessory protein 1 | 1 |
| **Kinase** | | |
| IPI00113606 | acylglycerol kinase | 4 |
| IPI00124444 | phosphofructokinase, platelet | 4 |
| IPI00387312 | phosphofructokinase, liver | 5 |
| IPI00554862 | phosphofructokinase, liver | 5 |
| IPI01027776 | Rho-associated, coiled-coil containing protein kinase 2 | 2 |
| IPI00116065 | serine/threonine kinase 24 | 2 |
| IPI00127596 | creatine kinase, muscle | 1 |
| IPI00661241 | phosphofructokinase, platelet | 1 |
| **Peptidase** | | |
| IPI00128945 | proteasome (prosome, macropain) subunit, beta type, 2 | 5 |
| IPI00761408 | lon peptidase 1, mitochondrial | 5 |
| IPI00136555 | YME1-like 1 ATPase | 1 |
| IPI00755442 | cathepsin A | 1 |
| IPI00845777 | lon peptidase 2, peroxisomal | 1 |
| IPI00125971 | proteasome (prosome, macropain) 26S subunit, ATPase, 6 | 3 |
| **Phosphatase** | | |
| IPI00109221 | SAC1 suppressor of actin mutations 1-like (yeast) | 5 |
| IPI00457898 | phosphoglycerate mutase 1 (brain) | 4 |
| **Transcription regulator** | | |
| IPI00896727 | cullin-associated and neddylation-dissociated 1 | 5 |
| IPI00652056 | metadherin | 2 |
| IPI00130409 | pre-mRNA processing factor 6 | 1 |
| **Translation regulator** | | |
| IPI00124287 | poly(A) binding protein, cytoplasmic 1 | 2 |
| IPI00274407 | Tu translation elongation factor, mitochondrial | 4 |
| IPI00318841 | eukaryotic translation elongation factor 1 gamma | 5 |
| IPI00466069 | eukaryotic translation elongation factor 2 | 5 |
| IPI00856453 | eukaryotic translation initiation factor 4 gamma, 1 | 2 |
| IPI00880750 | ribosomal protein S9 | 5 |
| IPI00457499 | GCN1 general control of amino-acid synthesis 1-like 1 (yeast) | 2 |
| IPI00460132 | Tu translation elongation factor, mitochondrial | 1 |
| IPI00474446 | eukaryotic translation initiation factor 2, subunit 1 alpha, 35kDa | 1 |
| IPI00128904 | poly(rC) binding protein 1 | 1 |

| | | |
|---|---|---|
| **Enzyme** | | |
| IPI00109109 | superoxide dismutase 2, mitochondrial | 5 |
| IPI00111218 | aldehyde dehydrogenase 2 family (mitochondrial) | 4 |
| IPI00111885 | ubiquinol-cytochrome c reductase core protein I | 5 |
| IPI00121105 | hydroxyacyl-CoA dehydrogenase | 2 |
| IPI00226430 | acetyl-CoA acyltransferase 2 | 5 |
| IPI00308882 | NADH dehydrogenase (ubiquinone) Fe-S protein 1, 75kDa (NADH-coenzyme Q reductase) | 5 |
| IPI00928416 | NADH dehydrogenase (ubiquinone) flavoprotein 1, 51kDa | 5 |
| IPI00117657 | optic atrophy 1 (autosomal dominant) | 5 |
| IPI00130624 | phospholipase D family, member 3 | 5 |
| IPI00131176 | cytochrome c oxidase subunit II | 5 |
| IPI00225201 | isoleucyl-tRNA synthetase | 3 |
| IPI00225275 | phosphorylase, glycogen, muscle | 3 |
| IPI00315794 | cytochrome b5 type B (outer mitochondrial membrane) | 4 |
| IPI00458208 | hydroxysteroid (17-beta) dehydrogenase 12 | 1 |
| IPI00556857 | dynamin 1-like | 5 |
| IPI00650049 | ALG2, alpha-1,3/1,6-mannosyltransferase | 3 |
| IPI00653158 | acetyl-CoA acyltransferase 2 | 5 |
| IPI00754992 | ribophorin II | 5 |
| IPI00755231 | CDP-diacylglycerol synthase (phosphatidate cytidylyltransferase) 2 | 5 |
| IPI00776197 | lactate dehydrogenase B | 1 |
| IPI00845772 | cytochrome c-1 | 5 |
| IPI00856866 | ribosomal protein S3 | 5 |
| IPI00875833 | methylenetetrahydrofolate dehydrogenase (NADP+ dependent) 1-like | 1 |
| IPI00928007 | myosin VA (heavy chain 12, myoxin) | 5 |
| IPI00929864 | cytochrome b | 5 |
| IPI00127172 | DEAD (Asp-Glu-Ala-Asp) box helicase 1 | 5 |
| IPI00122743 | aspartyl-tRNA synthetase | 5 |
| IPI00128400 | cytochrome P450, family 4, subfamily A, polypeptide 11 | 1 |
| IPI00222457 | aspartyl-tRNA synthetase | 4 |
| IPI00314673 | ST3 beta-galactoside alpha-2,3-sialyltransferase 4 | 1 |
| IPI00315488 | arginyl-tRNA synthetase | 3 |
| IPI00321308 | alanyl-tRNA synthetase | 1 |
| IPI00331628 | hydroxysteroid (17-beta) dehydrogenase 4 | 2 |
| IPI00403058 | glucosidase, alpha; neutral AB | 4 |
| IPI00404031 | peroxiredoxin 6 | 1 |
| IPI00415517 | mitofusin 2 | 1 |
| IPI00475343 | ALG5, dolichyl-phosphate beta-glucosyltransferase | 1 |
| IPI00624289 | large 60S subunit nuclear export GTPase 1 | 1 |
| IPI00625955 | acyl-CoA synthetase long-chain family member 6 | 2 |
| IPI00751087 | dolichyl-phosphate mannosyltransferase polypeptide 1, catalytic subunit | 1 |
| IPI00757748 | RAB6A, member RAS oncogene family | 1 |
| IPI00785470 | hydroxysteroid (17-beta) dehydrogenase 12 | 2 |
| IPI00831057 | phosphorylase, glycogen, muscle | 1 |
| IPI00858077 | aldehyde dehydrogenase 2 family (mitochondrial) | 1 |

| | | |
|---|---|---|
| IPI00882024 | membrane-associated ring finger (C3HC4) 5 | 2 |
| IPI00886151 | enoyl CoA hydratase, short chain, 1, mitochondrial | 1 |
| IPI00895397 | phosphatidylinositol glycan anchor biosynthesis, class T | 2 |
| IPI00915057 | acyl-CoA synthetase long-chain family member 3 | 1 |
| IPI00990118 | 3-hydroxy-3-methylglutaryl-CoA reductase | 1 |
| IPI01007839 | HECT and RLD domain containing E3 ubiquitin protein ligase 2 | 1 |
| IPI00123878 | DEAD (Asp-Glu-Ala-Asp) box polypeptide 39A | 2 |
| **Other** | | |
| IPI00762919 | huntingtin interacting protein 1 | 1 |
| IPI00112986 | BC1 (ubiquinol-cytochrome c reductase) synthesis-like | 3 |
| IPI00123342 | hypoxia up-regulated 1 | 5 |
| IPI00123494 | proteasome (prosome, macropain) 26S subunit, non-ATPase, 2 | 5 |
| IPI00136984 | ribosomal protein S7 | 3 |
| IPI00267295 | proteasome (prosome, macropain) 26S subunit, non-ATPase, 1 | 5 |
| IPI00313222 | ribosomal protein L6 | 4 |
| IPI00320241 | DnaJ (Hsp40) homolog, subfamily B, member 11 | 2 |
| IPI00331092 | ribosomal protein S4, Y-linked 1 | 5 |
| IPI00402981 | ribosomal protein S24 | 1 |
| IPI00420706 | leucine-rich pentatricopeptide repeat containing | 4 |
| IPI00648121 | ribosomal protein L23 | 1 |
| IPI00656269 | tyrosine 3-monooxygenase/tryptophan 5-monooxygenase activation protein, theta | 4 |
| IPI00750217 | autophagy related 9A | 5 |
| IPI00750847 | reticulon 3 | 1 |
| IPI00753044 | actin related protein 2/3 complex, subunit 3, 21kDa | 5 |
| IPI00754142 | mitochondrial carrier 2 | 5 |
| IPI00762507 | IQ motif and Sec7 domain 1 | 3 |
| IPI00807971 | ribosomal protein L18a | 5 |
| IPI00830211 | ribosomal protein S10 | 5 |
| IPI00857345 | ribosomal protein S5 | 5 |
| IPI00858004 | dynactin 1 | 2 |
| IPI00867847 | kinesin heavy chain member 2A | 3 |
| IPI00875638 | inner membrane protein, mitochondrial | 5 |
| IPI00880689 | ribosomal protein L24 | 4 |
| IPI00881758 | ribosomal protein S16 | 2 |
| IPI00889265 | neurochondrin | 5 |
| IPI00108143 | heterogeneous nuclear ribonucleoprotein H2 (H') | 5 |
| IPI00116372 | myeloid leukemia factor 2 | 2 |
| IPI00480414 | reticulon 3 | 3 |
| IPI00648723 | stathmin 1 | 1 |
| IPI00109082 | defender against cell death 1 | 2 |
| IPI00112947 | keratin 19 | 1 |
| IPI00124954 | kinesin family member 5B | 1 |
| IPI00127446 | cytohesin 1 interacting protein | 1 |
| IPI00129276 | eukaryotic translation initiation factor 3, subunit A | 2 |
| IPI00134137 | ganglioside induced differentiation associated protein 1 | 3 |
| IPI00230660 | ribosomal protein S15a | 2 |

| | | | |
|---|---|---|---|
| | IPI00330146 | nuclear fragile X mental retardation protein interacting protein 2 | 2 |
| | IPI00404595 | leucine rich repeat containing 8 family, member C | 1 |
| | IPI00404630 | TRK-fused gene | 1 |
| | IPI00406693 | astrotactin 2 | 2 |
| | IPI00420806 | dynein, cytoplasmic 1, light intermediate chain 2 | 1 |
| | IPI00463282 | keratin 2 | 4 |
| | IPI00469236 | lysophosphatidylglycerol acyltransferase 1 | 1 |
| | IPI00620460 | astrotactin 2 | 2 |
| | IPI00622240 | keratin 2 | 1 |
| | IPI00649033 | glial fibrillary acidic protein | 1 |
| | IPI00649324 | RAP1 GTPase activating protein | 1 |
| | IPI00649807 | proteasome (prosome, macropain) 26S subunit, non-ATPase, 3 | 5 |
| | IPI00652882 | vacuolar protein sorting 51 homolog (S. cerevisiae) | 3 |
| | IPI00653905 | saccharopine dehydrogenase (putative) | 1 |
| | IPI00762615 | pleckstrin and Sec7 domain containing | 2 |
| | IPI00775915 | ribosomal protein L10 | 5 |
| | IPI00831521 | FYVE, RhoGEF and PH domain containing 1 | 1 |
| | IPI00874955 | KIAA0368 | 1 |
| | IPI00875470 | proteasome (prosome, macropain) 26S subunit, non-ATPase, 12 | 1 |
| | IPI00880403 | transmembrane protein 30A | 2 |
| | IPI00970180 | developmentally regulated GTP binding protein 1 | 1 |
| | IPI00988654 | myosin XVIIIA | 2 |
| | IPI01007983 | pellino E3 ubiquitin protein ligase family member 2 | 3 |
| | IPI01027716 | ribosomal protein S18 | 1 |
| | IPI00115366 | kinesin family member 15 | 1 |
| | IPI00124264 | annexin A11 | 3 |
| | IPI00125454 | DnaJ (Hsp40) homolog, subfamily A, member 4 | 1 |
| | IPI00877240 | annexin A11 | 3 |
| **Extracellular space** | **Transporter** | | |
| | IPI00762198 | hemoglobin, beta adult major chain | 5 |
| | IPI00131695 | albumin | 2 |
| | IPI00315463 | receptor accessory protein 5 | 1 |
| | IPI00649772 | apolipoprotein H (beta-2-glycoprotein I) | 1 |
| | IPI00754566 | tubulointerstitial nephritis antigen-like 1 | 1 |
| | **Kinase** | | |
| | IPI00604969 | titin | 1 |
| | IPI00749707 | titin | 3 |
| | IPI00758400 | titin | 1 |
| | **Peptidase** | | |
| | IPI00126316 | carboxypeptidase A3 (mast cell) | 1 |
| | IPI00480212 | mannan-binding lectin serine peptidase 2 | 1 |
| | **Translation regulator** | | |
| | IPI00387389 | mitochondrial ribosomal protein L18 | 1 |
| | **Enzyme** | | |
| | IPI00113427 | lysozyme | 1 |

| | | |
|---|---|---|
| IPI00230212 | glutathione S-transferase mu 5 | 1 |
| IPI00649450 | glutathione S-transferase mu 5 | 4 |
| IPI00223446 | laminin, alpha 4 | 1 |
| IPI00988150 | oviductal glycoprotein 1, 120kDa | 1 |
| IPI01027598 | DDHD domain containing 1 | 1 |
| **Other** | | |
| IPI00124499 | keratin 79 | 5 |
| IPI00131995 | hyaluronan and proteoglycan link protein 1 | 5 |
| IPI00229829 | serpin peptidase inhibitor, clade A (alpha-1 antiproteinase, antitrypsin), member 11 | 2 |
| IPI00266188 | cofilin 2 (muscle) | 1 |
| IPI00380338 | immunoglobin superfamily, member 21 | 5 |
| IPI00554878 | copine family member IX | 3 |
| IPI00608077 | myosin, heavy chain 14, non-muscle | 2 |
| IPI00623371 | brevican | 5 |
| IPI00751005 | septin 8 | 5 |
| IPI00122015 | family with sequence similarity 49, member B | 2 |
| IPI00131674 | RIKEN cDNA 2210010C04 gene | 4 |
| IPI00136712 | olfactomedin 1 | 1 |
| IPI00225046 | olfactomedin 1 | 4 |
| IPI00109588 | collagen, type IV, alpha 1 | 1 |
| IPI00127100 | cochlin | 1 |
| IPI00223384 | polycystic kidney and hepatic disease 1 (autosomal recessive)-like 1 | 1 |
| IPI00225865 | N-acetyltransferase 14 (GCN5-related, putative) | 1 |
| IPI00229184 | hyaluronan and proteoglycan link protein 4 | 2 |
| IPI00229377 | RIKEN cDNA 4921501E09 gene | 1 |
| IPI00271262 | murinoglobulin 1 | 1 |
| IPI00309177 | thyroglobulin | 1 |
| IPI00330068 | mucin 6, oligomeric mucus/gel-forming | 1 |
| IPI00330653 | cartilage acidic protein 1 | 1 |
| IPI00341267 | leucine rich repeat and Ig domain containing 2 | 2 |
| IPI00459477 | latent transforming growth factor beta binding protein 1 | 1 |
| IPI00468360 | cilia and flagella associated protein 57 | 1 |
| IPI00648224 | collagen, type XV, alpha 1 | 1 |
| IPI00753106 | laminin, alpha 3 | 1 |
| IPI00762325 | absent in melanoma 1 | 1 |
| IPI00857149 | additional sex combs like transcriptional regulator 2 | 2 |
| IPI00875724 | family with sequence similarity 178, member A | 1 |
| IPI00876542 | serpin peptidase inhibitor, clade A (alpha-1 antiproteinase, antitrypsin), member 11 | 1 |
| IPI00885261 | polycystic kidney and hepatic disease 1 (autosomal recessive)-like 1 | 1 |
| IPI00928188 | TBC1 domain family, member 14 | 1 |
